# PI3Kδ contributes to ER stress-associated asthma through ER-redox disturbances: the involvement of the RIDD–RIG-I–NF-κB axis

**DOI:** 10.1038/emm.2017.270

**Published:** 2018-02-16

**Authors:** Hyun-Kyoung Kim, Geum-Hwa Lee, Kashi Raj Bhattarai, Raghu Patil Junjappa, Hwa-Young Lee, Mallikarjun Handigund, Anu Marahatta, Bidur Bhandary, In-Hwan Baek, Jae Sung Pyo, Hye-Kyung Kim, Ok Hee Chai, Hyung-Ryong Kim, Yong-Chul Lee, Han-Jung Chae

**Affiliations:** 1 Department of Pharmacology and Institute of New Drug Development, School of Medicine, Chonbuk National University, Jeonju, Republic of Korea; 2 College of Pharmacy, Kyungsung University, Busan, Republic of Korea; 3 Department of Anatomy, School of Medicine, Chonbuk National University, Jeonju, Republic of Korea; 4 Daegu Gyeonbuk Institute of Science & Technology (DGIST) Graduate School, Daegu, Republic of Korea; 5 Department of Internal Medicine, School of Medicine, Chonbuk National University, Jeonju, Republic of Korea

## Abstract

Hyperactivation of phosphoinositol 3-kinase (PI3K) has been suggested to be a potential mechanism for endoplasmic reticulum (ER) stress-enhanced airway hyperresponsiveness, and PI3K inhibitors have been examined as asthma therapeutics. However, the regulatory mechanism linking PI3K to ER stress and related pathological signals in asthma have not been defined. To elucidate these pathogenic pathways, we investigated the influence of a selective PI3Kδ inhibitor, IC87114, on airway inflammation in an ovalbumin/lipopolysaccharide (OVA/LPS)-induced asthma model. In OVA/LPS-induced asthmatic mice, the activity of PI3K, downstream phosphorylation of AKT and activation of nuclear factor-κB (NF-κB) were all significantly elevated; these effects were reversed by IC87114. IC87114 treatment also reduced the OVA/LPS-induced ER stress response by enhancing the intra-ER oxidative folding status through suppression of protein disulfide isomerase activity, ER-associated reactive oxygen species (ROS) accumulation and NOX4 activity. Furthermore, inositol-requiring enzyme-1α (IRE1α)-dependent degradation (RIDD) of IRE1α was reduced by IC87114, resulting in a decreased release of proinflammatory cytokines from bronchial epithelial cells. These results suggest that PI3Kδ may induce severe airway inflammation and hyperresponsiveness by activating NF-κB signaling through ER-associated ROS and RIDD–RIG-I activation. The PI3Kδ inhibitor IC87114 is a potential therapeutic agent against neutrophil-dominant asthma.

## Introduction

Allergic asthma, one of the most common respiratory diseases, is characterized by chronic airway inflammation, reversible airway obstruction, increased mucus production and nonspecific airway hyperresponsiveness.^1^ The ovalbumin/lipopolysaccharide (OVA/LPS)-induced murine asthma model is widely used to study the mechanisms of inflammation-related airway airway hyperresponsiveness and remodeling.^2, 3^ It has been suggested that airway inflammation in asthma is related to Toll-like receptor 4 signaling.^3^ Activation of Toll-like receptor 4 stimulates the phosphoinositide 3-kinase (PI3K)/Akt pathway as well as downstream nuclear factor-κB (NF-κB) signaling,^4^ resulting in the upregulation of proinflammatory chemokines.^3^ Signaling pathways involving the PI3Kδ isoform regulate lymphocyte development, differentiation and activation.^5^ Moreover, mast cell activation, differentiation and survival are related to PI3K p110δ catalytic activity.^6^ Blockade of PI3δK as a therapeutic strategy against severe asthma was initially accomplished through development of the quinazolinone purine series of inhibitors, as exemplified by 2-[(6-aminopurin-9-yl)methyl]-5-methyl-3-(2-methylphenyl)quinazolin-4-one (IC87114; ICOS Corporation, Bothell, WA, USA).^7^

The endoplasmic reticulum (ER) is an oxidative compartment that enables the formation of protein disulfide bonds by thioloxidation.^8^ Disulfide bond formation in the ER by protein disulfide isomerase (PDI) generates hydrogen peroxide (H_2_O_2_) that in turn forms highly reactive hydroxyl radicals in the presence of iron. Under conditions of PI3K hyperactivity, the ER is subjected to a high load of protein generation and is subsequently disturbed.^9^ In addition, the ER contains nicotinamide adenine dinucleotide phosphate oxidase (NOX), a major generator of superoxide.^10^ It has also been reported that NOX4 expression is increased in primary bronchial epithelial cells of patients with neutrophilic asthma, leading to increased production of ER reactive oxygen species (ROS).^11^ Thus, ER protein overload leading to a disturbance of ER redox homeostasis and accumulation of both misfolded proteins and ROS may be key pathogenic mechanisms in neutrophilic asthma.

Neutrophil-dominant asthma associated with ER stress caused by accumulation of misfolded proteins or ROS may also depend on activation of the transcription factor NF-κB.^12^ Accumulation of misfolded proteins induces an adaptive unfolded protein response (UPR). The inositol-requiring enzyme 1 (IRE1) is an initiator of one branch of the UPR that is closely linked to inflammatory signaling through the splicing of XBP1 (sXBP1).^13^ Moreover, it has been suggested that the IRE1-regulated IRE-1α-dependent decay (RIDD)–retinoic-acid inducible gene 1 (RIG-I) pathway regulates innate immunity against the potent mucosal pathogen *Vibrio cholera*, as well as against nucleic acids in the cytosol, affecting adaptive immunity.^14, 15^ Hyperphosphorylation of IRE1α increases RNase activity to degrade endogenous mRNA, a process termed ‘regulated IRE1α-dependent decay (RIDD) of mRNA’. The RIDD reaction produces single-strand mRNA fragments, activating RIG-I to cause a cell-autologous inflammatory response via the NF-κB and IFN pathways.^14, 16^ However, RIDD has not been studied in bronchial epithelial and immune cells from the murine OVA/LPS-induced severe asthma model.

In this study, we demonstrate enhanced PI3K/Akt signaling in the OVA/LPS-induced refractory asthma murine model and suppression by IC87114 attributable to the control of redox-sensitive PDI activity as well as related ROS and ER stress.

## Materials and methods

### Materials

Primary antibodies targeting the following proteins were used in this study: p-eIF2α, IRE-1α, CHOP (Cell Signaling Technologies, Danvers, MA, USA), GRP78, ATF6α, PERK, p-PERK, sXBP-1, β-actin (Santa Cruz Biotechnologies, Santa Cruz, CA, USA), p-IRE-1α (Abcam, Cambridge, MA, USA) and monoclonal PDI (clone 1D3, Enzo Life Sciences, Farmingdale, NY, USA). Horseradish peroxidase-conjugated secondary antibodies were obtained from Santa Cruz Biotechnology. IC87114 was obtained from Calbiochem (San Diego, CA, USA). Ovalbumin, lipopolysaccharide, N-ethylmaleimide (NEM), 4-hydroxy-2-nonenal (4-HNE) and dihydroethidine (DHE) were purchased from Sigma-Aldrich (St Louis, MO, USA).

### Mice

All experiments were performed using adult female inbred C57BL/6 mice (8−10 weeks old, 20−23 g) purchased from Damul Sciences (Dajeon, Korea).^17, 18^ Mice were housed (*n*=5 mice per cage) in a fully climate-controlled room at constant temperature and humidity on a 12 h:12 h light/dark cycle with food and water provided *ad libitum*. The animal ethics committee of Chonbuk National University of Korea approved all experiments (CBU 2013-0025) on the basis of the 3Rs (replacement, refinement and reduction). Animals were deeply anesthetized with isoflurane before killing via blood collection. All efforts were made to minimize the number of animals used and their suffering. Animal studies are reported in compliance with the ARRIVE guidelines.^19, 20^

### Group size, randomization and blinding

Mice were selected from the eligible pool and randomly divided into three groups of six for either sham induction using saline (SAL) challenge (control group) or asthma model induction with OVA/LPS plus vehicle or IC87114 treatment (OVA/LPS+IC87114 groups). The researchers treating the animals were not aware of the pharmacological treatments of each group.

### A protocol for murine models of asthma

The protocols and schedules used for saline control (SAL) and the two OVA/LPS groups (either IC87114 or vehicle administration) are summarized in Supplementary Figure 1A. All OVA/LPS group mice were sensitized intranasally with 75 μg of OVA plus 10 μg of LPS on days 0, 1, 2, 3 and 7 and were then challenged with 50 μg of OVA alone on days 14, 15, 21 and 22. On day 22 (3 h after the last airway challenge with OVA), IC87114 at 1 mg kg^−1^ or vehicle (0.05% dimethyl sulfoxide) diluted in 0.9% NaCl was administered in a volume of 30 μl by an intratracheal nonsurgical method.^21^

### Nonsurgical intratracheal method

Model mice received a single dose of IC87114 (1 mg kg^−1^) or vehicle intratracheally by a nonsurgical method as previously described.^21^ We selected 1 mg kg^−1^ based on reports of the efficacy of this dose for the treatment of pulmonary disease.^22^ Using forceps, the tongue was pulled out and held to the side. With the other hand, a 1 ml syringe containing the drug or vehicle, fitted with a bent gavage needle, was inserted into the trachea and the plunger depressed to deliver the drug. Venous blood samples were collected at the indicated time points described in Supplementary Figure 1A. Six animals were killed at each time point for subsequent analyses.

### Measurement of airway hyperreactivity

Airway hyperreactivity was assessed by whole-body plethysmography during airflow obstruction induced by a methacholine (MeCh) aerosol.^23, 24^ Each group of mice was exposed for 3 min to aerosolized saline, followed by exposure to increasing concentrations of aerosolized MeCh (0, 6.25, 12, 25 and 50 mg ml^−1^) dissolved in isotonic saline. Each dose was nebulized for 2 min, and airway responses were recorded for 5 min. Following each administration through the inlet of the main chamber, the enhanced pause (Penh) was recorded for 3 min. The Penh values measured during each 3 min sequence were averaged for each dose. Penh data were plotted as the change from baseline per dose of MeCh. The percentage increases in Penh over baseline at each concentration were used to compare airway reactivity among the experimental groups.

### Analysis of BALF samples

Bronchoalveolar lavage fluid (BALF) samples (1 ml) were obtained from each mouse. Samples were centrifuged (600 *g*, 3 min), and the supernatant was stored at –20 °C for cytokine analysis. The cell pellets from samples were pooled for total cell counts using a Model Z1 (Beckman-Coulter, Miami, FL, USA) after lysis of erythrocytes (Zap-Oglobin II, Beckman-Coulter, Fullerton, CA, USA). Slides were loaded with cells, centrifuged (700 × *g*, 3 min) and stained with Diff-Quick (Baxter, Detroit, MI, USA). Differentials were enumerated via light microscopy.

### PI3K kinase assay

The *in vitro* PI3K activity was determined using a commercially available enzyme-linked immunosorbent assay kit (Echelon Biosciences, Salt Lake City, UT, USA). Briefly, lung tissues were lysed via addition of 1% CHAPS, 150 mM NaCl, 20 mM Tris-HCl, 10 mM HEPES, phosphatase inhibitor (Sigma-Aldrich) and protease inhibitors (Roche, Palo Alto, CA, USA). The lysates were centrifuged at 13 000 r.p.m. for 30 min at 4 °C. The supernatant protein concentration was measured by the Bradford assay, and immunoprecipitation was performed with an anti-PI3K p85 antibody (Santa Cruz Biotechnology) and protein A/G beads (Sigma-Aldrich) from 500 μg of the tissue lysate protein. The activity of the recovered sample was assayed according to the manufacturer’s instructions.

### Subcellular fractionation

Subcellular extractions (cytosol, nuclear and ER) were performed as previously described.^25^ Lung tissues were resuspended in osmotic buffer (0.32 M sucrose, 1 mM MgCl_2_, 10 mM Tris-HCl, pH 7.4) and lysed by 20 passes with a Dounce homogenizer. The homogenate was centrifuged at 1000 × *g* for 10 min at 4 °C to obtain the nuclear fraction (pellet). The supernatant was then centrifuged at 13 000 × *g* for 30 min at 4 °C. The second supernatant was centrifuged for 1 h at 100 000 × *g* at 4 °C using a SW32.1 rotor in an L8-80M ultracentrifuge (Beckman Coulter) to obtain the cytosol (supernatant) and ER (pellet) fractions. Fractions were collected and stored at −80 °C until use.

### Quantitative real-time PCR

The mRNA expression levels were determined by quantitative real-time PCR (qRT-PCR) as previously described.^22^ Total RNA was isolated from lung tissue with Trizol reagent (Invitrogen Life Technologies, Carlsbad, CA, USA), and complementary DNA was synthesized from RNA using a PrimeScript Reverse Transcriptase kit according to the manufacturer’s protocols. Quantitative real-time PCR was performed using SYBR Green PCR Master Mix (Applied Biosystems, Foster City, CA, USA) according to the protocols provided by the manufacturer with an ABI prism 7700 Sequence Detector System (Applied Biosystems). Murine primers used in this study are listed in Supplementary Table 1. Target gene mRNA expression levels were calculated using the ΔCt method and normalized to glyceraldehyde 3-phosphate dehydrogenase mRNA expression.

### Immunohistochemistry and periodic acid–Schiff staining

Histological studies were performed as previously described.^7^ Lung tissues were fixed in a 4% formalin solution for 24 h, embedded in paraffin and stained with antibodies or periodic acid–Schiff reagent as previously described.^26^ Serial 4 μm thick sections were prepared, blocked with 5% serum after antigen retrieval and incubated at 4 °C overnight with one of the following primary antibodies: anti-pS6K, anti-4E-BP1, Mucin5AC or 4-HNE. Immunostaining was visualized with 3,3’-diaminobenzidine, and sections were counterstained with hematoxylin and eosin.

### Western blotting assay

The protein levels were determined by western blotting analysis as previously described.^7^ Lung tissues were lysed in RIPA buffer (10 mM Tris pH 7.4, 150 mM NaCl, 1% Triton X-100, 1% sodium deoxycholate, 0.1% sodium dodecyl sulfate, 1 mM phosphatase inhibitor cocktail, 1 mM protease inhibitor cocktail) for 30 min on ice. Equal amounts of protein were separated on 10% SDS–polyacrylamide gel electrophoresis gels and transferred to polyvinylidene difluoride membranes using a Bio-Rad mini-transfer tank. Membranes were probed with the indicated primary antibodies. After incubation with the secondary antibody, blots were developed using a chemiluminescence detection system. Images were acquired and analyzed with ImageJ software (U. S. National Institutes of Health, Bethesda, MD, USA). The protein expression levels (band intensities) were normalized to those of β-actin.

### Enzyme-linked immunosorbent assay

The concentrations of interleukin (IL)-4, IL-5, IL-13 and IL-17 in BALF were measured via individual enzyme-linked immunosorbent assay kits according to the manufacturer’s instructions (BD Biosciences, San Jose, CA, USA).

### Lipid peroxidation assay

Lipid peroxidation was assessed using a lipid hydroperoxide assay kit purchased from Cayman Chemicals (Ann Arbor, MI, USA). Lung microsomes (1 mg) were homogenized in 1 ml of ice-cold 2% SDS buffer. The sample homogenates, as well as malondialdehyde standards, were incubated with SDS and 0.8% thiobarbituric acid (20% acetic acid, pH 3.5) in the presence of 0.8% butylated hydroxytoluene at 95 °C for 1 h. After incubation, samples were cooled on ice and centrifuged at 3000 r.p.m. for 15 min. Peroxidation levels in supernatants were assessed on a spectrophotometer at an absorbance of 532 nm.

### GSH/GSSG (reduced/oxidized glutathione) ratio assay

Oxidative stress in the lung was examined using a glutathione assay kit from Cayman Chemicals according to the manufacturer’s instructions.^27^

### Oxy blot assay

Oxidative protein carbonylation assays were performed on lung tissue following western blot using an OxyBlot Protein Detection Kit (Millipore, Billerica, MA, USA) according to the manufacturer’s instructions. The carbonyl groups in the protein side chains were derivatized to DNP-hydrazone by reaction with 2,4-dinitrophenylhydrazine (DNPH) following the manufacturer’s instructions. After derivatization of the protein sample, one-dimensional electrophoresis was carried out on 10% SDS polyacrylamide gel electrophoresis gels. Proteins were transferred to polyvinylidene difluoride membranes. After incubation with an anti-DNP antibody, the blot was developed using a chemiluminescence detection system.^28^

### PDI redox state and high-molecular-weight protein complex formation

Procedures were performed as previously described.^26^ Briefly, ER fractions were incubated with or without 10 mM dithiothreitol (DTT) for 10 min at 37 °C or 5 mM diamide as a redox state control. The ER fractions were washed twice with ice-cold phosphate-buffered saline supplemented with 20 mM NEM and lysed in buffer (20 mM Tris, pH 7.4; 150 mM NaCl; and 1% Triton X-100) for 30 min on ice. The lysate was cleared by centrifugation, and the protein concentrations were determined by the Bio-Rad Bradford protein assay. Then, 20 μg of total protein per gel lane was separated on nonreducing gels (no DTT, not boiled) using 12% polyacrylamide to distinguish the redox forms of PDI and 8% polyacrylamide to detect complexes between PDI and its substrate proteins.

### Detection of carbonylated PDI

Detection of carbonylated PDI was performed as previously described.^26^ Proteins (100 μg) from lung tissue homogenates were derivatized for 5 min using the OxyBlot kit (Millipore). The reaction was stopped by the addition of a neutralization solution according to the manufacturer’s instructions. To remove DNPH, proteins were pelleted by ultracentrifugation at 100 000 *g* for 1 h at 4 °C. Protein pellets were washed with immunoprecipitation lysis buffer (20 mM Tris, pH 7.4; 150 mM NaCl; and 0.5% Triton X-100 and protease inhibitor) and resuspended in 300 μl of fresh immunoprecipitation lysis buffer. Resuspension solutions were incubated with PDI antibodies and then with protein A/G-Sepharose (50% slurry, Sigma-Aldrich) for an additional 2 h. Immunocomplexes were collected by centrifugation, washed three times with 10 mM Tris (pH 7.5) containing 0.1 M NaCl and 1% Triton X-100, eluted in 50 μl of sample buffer, and resolved on non-reducing gels, followed by western blotting with anti-DNP rabbit antibody according to the OxyBlot kit instructions.

### H_2_O_2_ measurement

Release of H_2_O_2_ from isolated ER was measured using the Amplex Red Hydrogen Peroxide/Peroxidase Assay (Life Technologies, Darmstadt, Germany) in assay buffer containing 115 mM KCl, 10 mM KH_2_PO_4_, 2 mM MgCl_2_, 3 mM HEPES, 1 mM EGTA, pH 7.2. The *in vitro* levels of H_2_O_2_ were measured according to the manufacturer’s protocol.

### *In vivo* siRNA transfection

*In vivo* small interfering RNA (siRNA) transfection was performed as previously described.^29, 30^ NF-κB and control siRNAs were purchased from Santa Cruz. siRNA was dissolved in a 5% glucose solution and *in vivo* jetPEI (Polyplus Transfection, New York, NY, USA) to an N/P ratio (the number of nitrogen residues of *in vivo*-jetPEI per DNA phosphate) of 7, and a total of 50 μl of the siRNA-jetPEI complex was administered intranasally to nude mice before the last challenge.

### Data normalization

As a control group, mice were sensitized and challenged intranasally with saline (SAL). We calculated the control mean and all of the values of all of the groups relative to the mean value of the control group; the control group value was set as 1 or 100%, and all of the individual values were expressed as fold changes relative to the control mean.^31^

### Data and statistical analysis

All data and statistical analyses complied with the recommendations for experimental design and analysis in pharmacology.^31^ Data are expressed as the mean±s.e.m. GraphPad Prism version 5.01 (GraphPad Software, San Diego, CA, USA) was used for all statistical analyses. Comparisons between two groups were made using Student’s unpaired *t*-test for normally distributed data or the Mann–Whitney *U*-test as the nonparametric equivalent. Comparisons between three or more groups were performed using one-way analysis of variance followed by Tukey’s *post hoc* test for normally distributed data or with a Kruskal–Wallis *H*-test for nonnormally distributed data. A threshold of *P*<0.05 was designated as statistically significant for all tests.

## Results

### OVA with LPS treatment induces airway inflammation and ER stress

**Figure 1:**
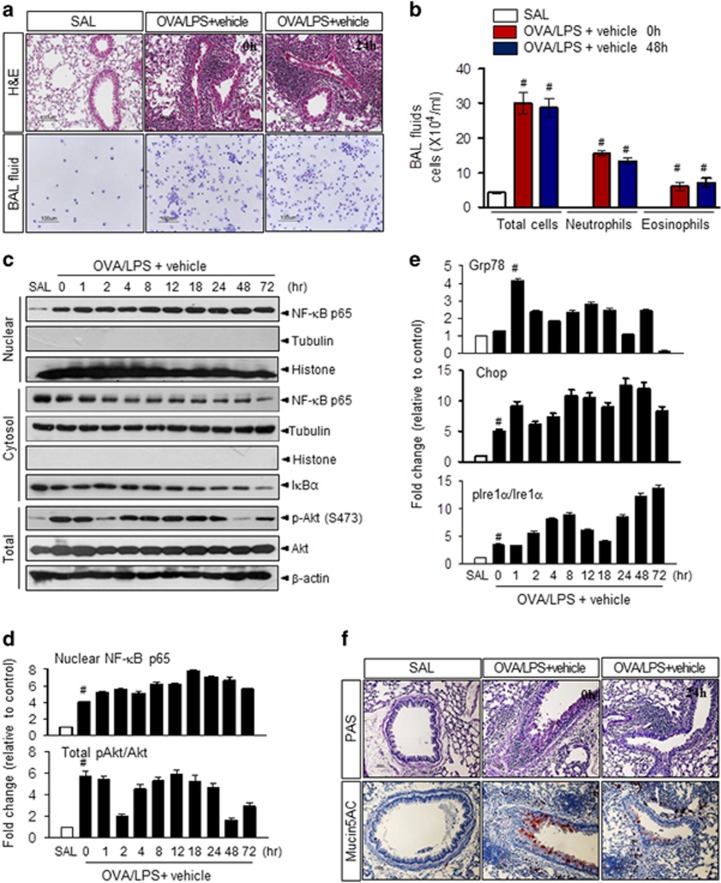
Ovalbumin (OVA) plus lipopolysaccharide (LPS) induces severe asthma in mice. (**a**) Lung tissues and bronchoalveolar lavage fluid (BALF) cells obtained from OVA/LPS-sensitized/challenged mice (OVA/LPS), saline/saline-sensitized/challenged mice (SAL) and OVA/LPS-sensitized/challenged mice administered drug and vehicle were stained with hematoxylin and eosin (upper) and Diff-Quick solution (lower). Magnification, × 100. (**b**) Total cells and differential cellular components of BALF (*n*=6 animals per experimental group. (**c**) Immunoblotting of nuclear factor-κB (NF-κB), IκBα and AKT proteins from samples collected by subcellular fractionation of lung tissues. (**d**) Changes in the NF-κB and AKT protein expression levels (mean±s.e.m.) relative to the control (SAL) group (*n*=6 animals per group). (**e**) Changes in endoplasmic reticulum (ER) stress marker protein expression (mean±s.e.m.) relative to the control (SAL) group (*n*=6 animals per group). (**f**) Lung sections from mice were prepared and stained with periodic acid–Schiff (PAS; upper) and anti-mucin5AC antibody (lower). Mice challenged with saline (SAL) were used as the control group. ^#^*P*<0.05 versus SAL.

To determine whether severe neutrophilic asthmatic inflammation is induced by OVA in combination with 10 μg of LPS in mice, we performed the experiments shown in Supplementary Figure 1a as described previously.^2^ Mice were sensitized intranasally with OVA plus LPS (OVA/LPS) on days 0, 1, 2 and 7 and then challenged intranasally with OVA alone on days 14, 15, 21 and 22 (hereafter referred to as OVA/LPS-treated mice). At 3 h after the last challenge, we administered vehicle (0.05% dimethyl sulfoxide) by nonsurgical intratracheal administration. As expected, OVA/LPS-treated mice exhibited increased total inflammatory cell counts in BALF, inflammatory cell infiltration in bronchioles and thickening of the airway epithelium compared with control (SAL) mice sensitized with saline, and these inflammatory responses persisted for up to 3 days after OVA challenge (Figures 1a and b). Phosphorylation of AKT increased in OVA/LPS-sensitized/OVA-challenged mice (Figure 1c). Moreover, NF-κB, a central transcription factor in the inflammatory response, was translocated into the nucleus simultaneously with degradation of the inhibitory factor IκBα (Figures 1c, d and Supplementary Figure 1b). The expression levels of the ER chaperone GRP78, p-PERK and its downstream effector eIF2α, p-IRE-1α and its downstream effector spliced XBP1 (sXBP-1) and cleaved ATF6, constituting the three branches of the adaptive UPR, were enhanced in OVA/LPS-treated mice compared with SAL controls (Figure 1e and Supplementary Figures 1c and d). Of the mucin genes in the adult human lung, muc5AC is the predominant gene expressed and is the most abundant in mucus secretions.^32^ Secretion of mucin5AC into BALF was elevated in OVA/LPS-treated mice, indicating that mucus is hyperproduced in this asthma model (Figure 1f). These results suggest that OVA/LPS treatment induces severe and generally neutrophil-dominant asthma accompanied by ER stress, NF-κB inflammatory signaling and airway remodeling.

### IC87114 attenuates OVA/LPS-induced airway inflammation and its signaling

**Figure 2:**
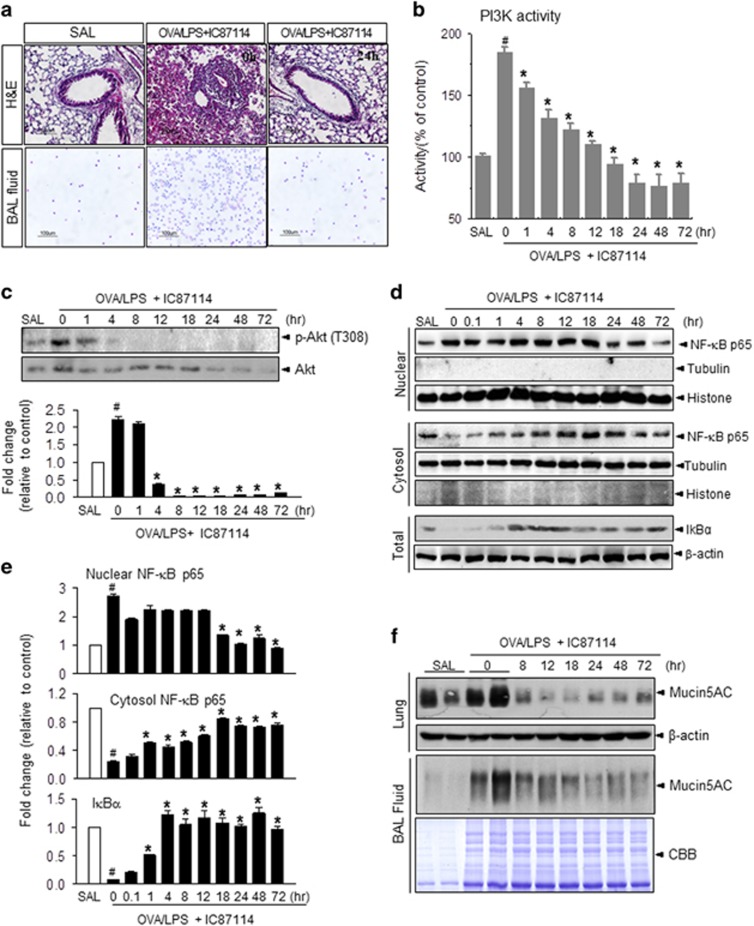
IC87114 attenuates airway inflammation in ovalbumin/lipopolysaccharide (OVA/LPS)-induced asthma. (**a**) Lung tissues and bronchoalveolar lavage fluid (BALF) cells obtained from OVA/LPS-treated mice, saline (SAL)-treated mice and OVA/LPS-treated mice administered 1 mg kg^−1^ IC87114 were stained with hematoxylin and eosin (upper) and Diff-Quick solution (lower). Magnification × 100. (**b**) Phosphoinositol 3-kinase (PI3K) activity was measured as described in the Materials and methods (*n*=6 animals per experimental group). (**c**) Immunoblotting and densitometric analyses (lower) were performed with an anti-p-AKT or AKT antibody. (**d**) Immunoblotting was performed for nuclear factor-κB (NF-κB) or IκBα after isolating nuclear, cytosolic, and total fractions. (**e**) Densitometric analyses of nuclear NF-κB p65/nuclear histone, cytosolic p65/cytosolic tubulin and IκBα/β-actin were performed. (**f**) Immunoblotting was performed with an anti-Mucin5AC antibody in lung tissues (upper) and in BALF (CBB staining for loading control). The lines with SAL and OVA/LPS represent two samples from the same conditions. In (**c**) and (**e**), values are presented as the mean (±s.e.m.) fold change relative to the control (SAL) group (*n*=6 animals per experimental group). Mice challenged with saline (SAL) were used as the control group. ^#^*P*<0.05 versus SAL; *P<0.05 versus OVA/LPS only.

To assess the possible therapeutic effects of IC87114, mice were treated with 1 mg kg^−1^ IC87114 at 3 h after the last OVA challenge by intratracheal administration and then killed at various times to assess the progression of pathology. Lungs from OVA/LPS-treated mice showed widespread peribronchiolar and perivascular inflammatory cell infiltrates compared with SAL mice, as shown in Figure 1 (Figure 2a). However, administration of IC87114 resulted in a significant reduction in inflammatory cell infiltration and recovery of airway morphological alterations (Figure 2a and Supplementary Figure 2). The increased numbers of total cells, eosinophils and neutrophils observed in OVA/LPS-treated mice that received vehicle were significantly decreased by IC87114 treatment (Supplementary Figures 3a–c and Figure 1a). Airway responses to MeCh were measured 1 h before and 0, 4 and 8 h after aspiration. At 1 h before aspiration, OVA/LPS-treated asthma groups showed airway hyperresponsiveness, as indicated by significantly increased Penh values in response to MeCh. In contrast, IC87114 exposure significantly reduced Penh values in OVA/LPS mice (Supplementary Figure 3d).

PI3K activity and downstream phosphorylation of the targets AKT, S6K and 4E-BP1 were also inhibited by administration of IC87114 (Figures 1c, 2b, c and Supplementary Figure 4). Subcellular fraction analysis showed that nuclear translocation of NF-κB and degradation of cytosolic IκBα were significantly inhibited by IC87114 treatment compared with OVA/LPS-treated mice that received vehicle (Figures 1c, 2d and e). In patients with high inflammation/airway remodeling status, mucin secretion is also considered a pathological phenomenon.^33^ Administration of IC87114 significantly inhibited the induction and secretion of mucin5AC compared with vehicle treatment, indicating that submucosal edema and mucus hypersecretion were reduced by IC87114 (Figures 1f and 2f).

We then assessed secretion of the T helper type 2 cytokines IL‐4, IL‐5, IL-10 and IL‐13, as overproduction of these factors also contributes to asthmatic conditions.^34^ The OVA/LPS treatment induced marked increases of the BALF IL-4, IL-5, IL-13 and IL-17 levels compared with SAL-treated mice (Supplementary Figure 5), and the levels of these cytokines were drastically reduced in a time-dependent manner by IC87114 administration compared with vehicle treatment. Consistent with this reduction in secretion, the IL-4, IL-5, IL-13 and IL-17 mRNA levels in lung tissues were decreased by IC87114 compared with vehicle (Supplementary Figure 6).

The role of NF-κB in this asthma model was further examined through genetic interference. NF-κB and control siRNA were dissolved in transfection reagents and administered intranasally to nude mice before the last challenge. As seen in Supplementary Figure 7, an increased number of total cells was observed in OVA/LPS-treated mice that received control siRNA, whereas these numbers were significantly decreased under NF-κB siRNA-administered conditions (Supplementary Figure 7a). Moreover, NF-κB translocation into the nucleus was reduced and the levels of IL-4, IL-5 and IL-13 were also reduced in NF-κB siRNA-treated mice compared with control siRNA-treated mice (Supplementary Figures 7b and c).

### IC87114 regulates the OVA/LPS-induced ER redox imbalance and associated ROS accumulation

**Figure 3:**
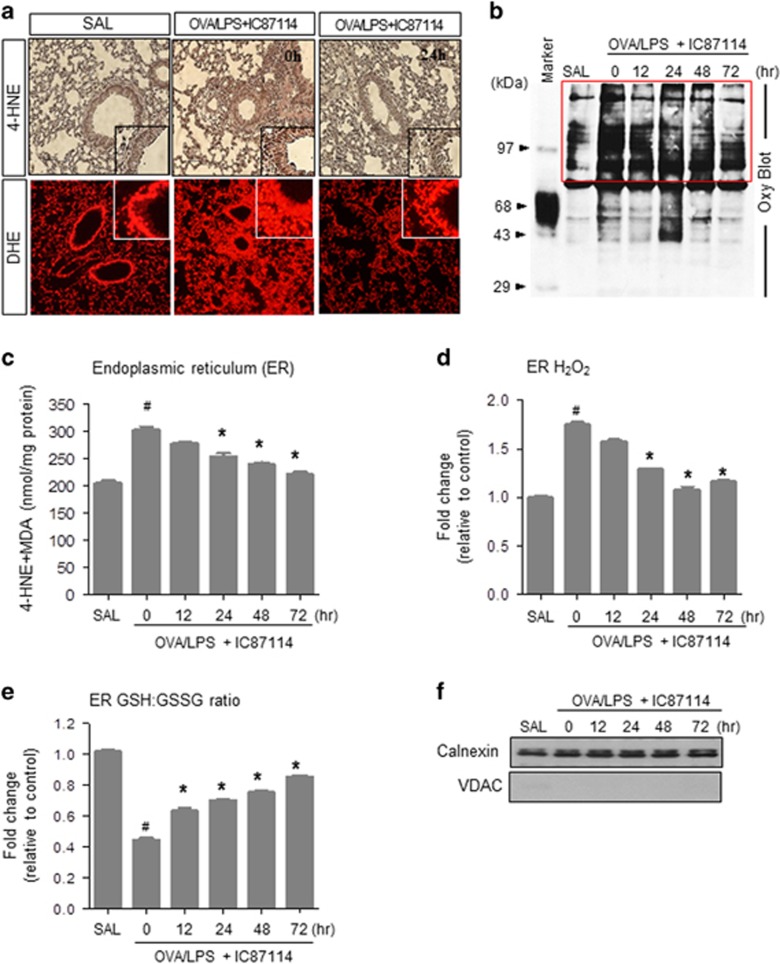
IC87114 attenuates stress-associated reactive oxygen species (ROS) accumulation in the endoplasmic reticulum (ER) of lung epithelial cells. (**a**) The 4-hydroxy-2-nonenal (4-HNE) and dihydroethidine hydrochloride (DHE) staining in lung tissues obtained from ovalbumin/lipopolysaccharide (OVA/LPS)-treated mice, saline (SAL)-treated mice and OVA/LPS-treated mice administered 1 mg kg^−1^ IC87114. (**b**) Lysates from lung tissues analyzed for the presence of oxidized proteins by oxyblot analysis. 4-HNE and malondialdehyde (MDA) (**c**) and hydrogen peroxide levels (**d**) in lung ER fractions from IC87114-treated mice. (**e**) GSH/GSSG ratio in the lung ER fraction. In (**d**) and (**e**), values are presented as the mean (±s.e.m.) fold changes relative to the control (SAL) group (*n*=6 animals per experimental group). (**f**) Immunoblotting was performed using an anti-calnexin antibody as an ER marker and an anti-voltage-dependent anion-selective channel (anti-VDAC) antibody as a mitochondrial marker, after isolating ER fractions. Mice challenged with saline (SAL) were used as the control group. ^#^*P*<0.05 versus SAL; *P<0.05 versus OVA/LPS only.

Oxidative stress is one of the cardinal pathogenic features of allergic lung inflammation, and hence we measured membrane lipid peroxidation, protein oxidation and glutathione redox status (GSH/GSSG balance) in lung tissue.^35^ To determine the oxidative stress caused by ER-derived ROS, ER fractions free of mitochondria were prepared and were immunoblotted for calnexin as an ER marker and voltage-dependent anion channel as a mitochondrial marker. As shown in Figure 3f, it was confirmed that pure ER fractions without mitochondria were collected.

Lipid peroxidation products were assessed by 4-HNE and DHE staining in lung sections. Peroxidation was induced by OVA/LPS treatment and reduced by IC87114 treatment (Figure 3a and Supplementary Figure 8a). An increase in protein oxidation was also detected in OVA/LPS-treated mice that was reduced in a time-dependent manner by IC87114 (Figure 3b and Supplementary Figure 8b). As the accumulation of oxidized proteins in the ER lumen generates ROS that leads to oxidative damage, lipid peroxidation pattern analysis was performed in the ER fraction. The levels of 4-HNE and malondialdehyde in the ER fraction were reduced by IC87114 treatment (Figure 3c). Moreover, the intra-ER hydrogen peroxide levels were also increased in OVA/LPS-sensitized/OVA-challenged mice and reduced by IC87114 (Figure 3d and Supplementary Figure 8c). The ER balance of GSH and GSSG is another parameter that reflects the ER protein oxidation status.^28^ The GSH/GSSG ratio was reduced in OVA/LPS-treated mice and normalized by IC87114 treatment (Figure 3e). Taken together, these results indicate that PI3δK hyperactivity is involved in the overloaded ER protein status and ROS accumulation of neutrophil-dominant asthma.

### IC87114 controls oxidative folding status through PDI and NOX4 in the ER

**Figure 4:**
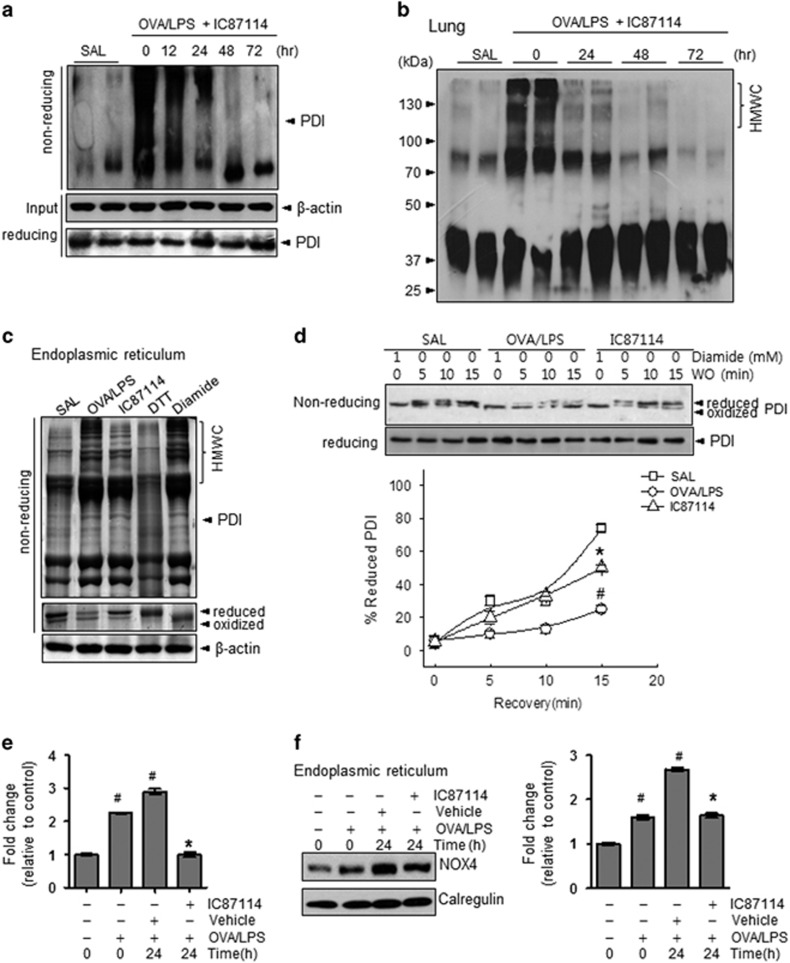
IC87114 attenuates the disturbances in protein disulfide isomerase (PDI) folding status and NOX4 expression. (**a**) Carbonylated proteins in the immunoprecipitates from an anti-PDI antibody on nonreducing gels. (**b**) High-molecular-weight complex formation was analyzed with an anti-PDI antibody. (**c**) Reduced and oxidized forms of PDI were analyzed as described in the Materials and methods. Endoplasmic reticulum (ER) lysates treated with 10 mM dithiothreitol (DTT) and 5 mM diamide for 15 min were used as standards for the reduced and oxidized forms of PDI, respectively. (**d**) Lung lysates were treated with or without 1 mM diamide for 15 min, and then after washout during the indicated time period, the reduced and oxidized forms of PDI were analyzed via immunoblotting. The mRNA (**e**) and protein expression levels of NOX4 (**f**) in lung tissues obtained from ovalbumin/lipopolysaccharide (OVA/LPS), saline (SAL) and OVA/LPS-treated mice administered 1 mg kg^−1^ IC87114 and vehicle (0.05% dimethyl sulfoxide (DMSO) with saline). The lines with SAL and OVA/LPS represent two samples of the same condition. In (**e**) and (**f**), values are presented as the mean (±s.e.m.) fold changes relative to the control (SAL) group (*n*=6 animals per experimental group). Mice challenged with saline (SAL) were used as the control group. ^#^*P*<0.05 versus SAL; *P<0.05 versus vehicle.

To examine the mechanisms of ER-associated ROS generation, we first measured PDI carbonylation. Oxidative stress results in the modification of protein side chains to carbonyl derivatives (aldehydes and ketones).^36^ Carbonylation of PDI was enhanced in OVA/LPS-induced asthma and significantly reversed by IC87114 treatment (Figure 4a). Under ER stress conditions, protein folding is compromised and multiprotein associations persist, resulting in the accumulation of high-molecular-weight complexes.^26^ The persistence of high-molecular-weight complexes may eventually lead to the formation of insoluble multiprotein complexes that further perturb ER function and predispose cells to apoptosis. Consistent with substantial PI3δK-dependent ER stress, mice with OVA/LPS-induced asthma exhibited increased formation of high-molecular-weight complexes that was prevented by IC87114 (Figures 4b, c and Supplementary Figure 8d). To confirm the PDI redox status, lung lysate samples were exposed to various concentrations of the reducing agent DTT or control washout after treatment with the oxidative agent diamide (Figure 4d and Supplementary Figure 9). The oxidized form of PDI was readily changed to the reduced form of PDI under DTT or control washout conditions with normal saline, but was relatively persistent under OVA/LPS-induced asthma conditions. The persistently oxidized form of PDI was reversed by IC87114 treatment under both DTT treatment or washout conditions.

ER-localized NOX4 physically interacts with PDI under intra-ER ROS accumulation.^37^ Moreover, NOX4 expression is increased in primary basal epithelial cells in asthma.^11^ We thus speculated that NOX4 may be involved in ER stress. Indeed, both the NOX4 mRNA and protein expression levels were increased in OVA/LPS-induced asthma and reversed by IC87114 treatment (Figures 4e and f). Collectively, our findings indicate that the OVA/LPS sensitizing/challenging process induces ER redox disturbances involving PDI–Ero1α redox imbalance and NOX4 overexpression.

### IC87114 regulates RIG-I signaling by controlling RIDD activity of IRE1α

**Figure 5:**
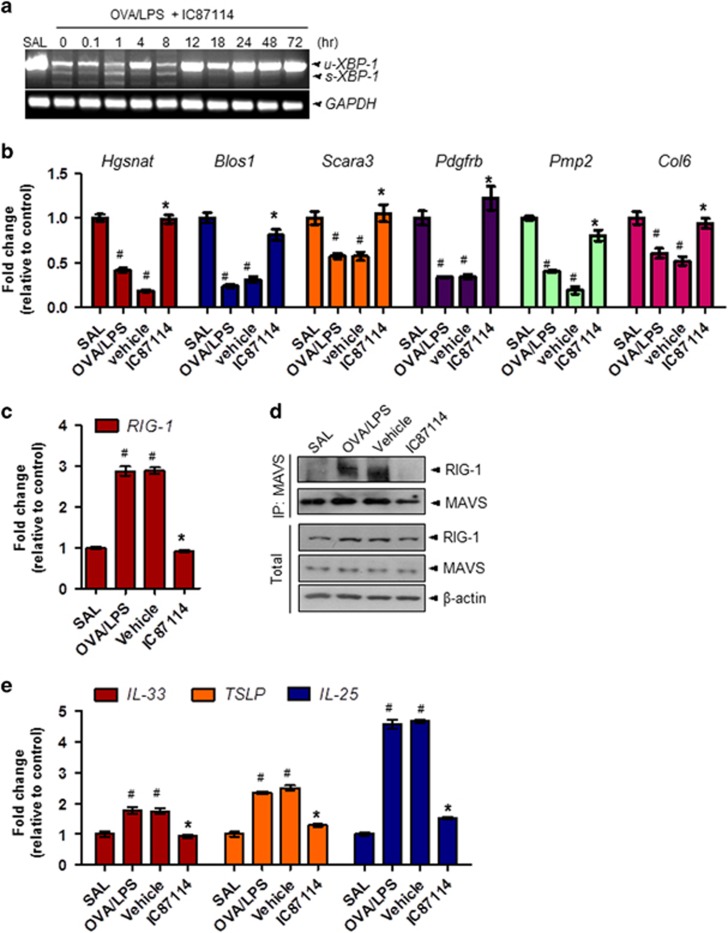
IC87114 reduces IRE-1α-dependent decay (RIDD) activity of inositol-requiring enzyme-1α (IRE1) and associated retinoic-acid inducible gene 1 (RIG-I) signaling in ovalbumin/lipopolysaccharide (OVA/LPS)-induced asthma. (**a**) Quantitative real-time PCR (qRT-PCR) analysis of *Xbp-1* at the indicated times in lung tissues from OVA/LPS, saline (SAL) and OVA/LPS-treated mice administered 1 mg kg^−1^ IC87114. The mRNA levels of the known IRE1 RIDD target genes *Hqsnat*, *Blos1*, *Scara3*, *Pdqfrb, Pmp2* and *Col6* (**b**), and RIG-I (**c**) were assessed by qRT-PCR. (**d**) Immunoblotting with antibodies against MAVS or RIG-I and immunoprecipitation (IP) were performed in lung lysates. (**e**) The mRNA levels of bronchial epithelium cytokines interleukin (IL)-25, IL-33 and thymic stromal lymphopoietin (TSLP) were assessed by qRT-PCR. In (**b**), (**c**) and (**e**), values are presented as the mean (±s.e.m.) fold changes relative to the control (SAL) group (*n*=6 animals per experimental group). *uXbp-1*, unspliced forms of *Xbp-1* mRNA; *sXbp-1*, spliced forms of *Xbp-1* mRNA. Mice challenged with saline (SAL) were used as the control group. ^#^*P*<0.05 versus SAL; *P<0.05 versus vehicle.

The three representative UPR signaling pathways, IRE1α/XBP-1, PERK/eIF2α and ATF6, were highly activated by OVA/LPS (Figure 1e and Supplementary Figures 1C, D); these signaling responses were time-dependently suppressed by IC87114 treatment (Supplementary Figure 10). Specifically, sXBP-1 mRNA was downregulated by IC87114 compared with vehicle (Figure 5a and Supplementary Figure 11). Furthermore, the expression levels of the known IRE1α–RIDD target genes *Hqsnat*, *Blos1*, *Scara3*, *Pdqfrb*, *Pmp2* and *Col6* were decreased in OVA/LPS-induced asthma and recovered with IC87114 administration (Figure 5b). RIG-I is a pattern recognition receptor that senses virus-derived RNA and self mRNA fragments, and IRE1α–RIDD exerts a variety of immune responses activated by binding to Mitochondrial antiviral-signaling protein (MAVS).^14^ To evaluate whether RIG-I signaling is activated by RIDD in OVA/LPS-induced asthma, we analyzed the expression levels of RIG-I using quantitative real-time PCR and immunoblotting. The expression levels of RIG-I mRNA and protein were increased by OVA/LPS and were significantly regulated by IC87114 (Figures 5c and d). Next, we performed immunoprecipitation assays in lung tissues to identify the association between RIG-I and MAVS. Complex formation between RIG-I and MAVS was clearly increased under OVA/LPS-treated conditions compared with the SAL condition that was significantly dissociated by IC87114 treatment.

Thus, RIG-I signaling activated by RIDD of IRE1α can also be regulated by IC87114. Moreover, the three upstream bronchial epithelial cytokines IL-33, thymic stromal lymphopoietin and IL-25 that were previously found to be increased in a viral-induced asthma exacerbation model through RIG-I pathway activation^38^ were also elevated by the RIG-I pathway in the OVA/LPS asthma model (Figure 5e). This was also reversed by IC87114. These results suggest that RIG-I signaling through IRE1α may be involved in the exacerbation of asthma. Furthermore, this process is dependent on PI3δK hyperactivation and is reversible by IC87114 treatment.

## Discussion

In this report, we present our initial preclinical findings on the development of aerosolized PI3Kδ inhibitors for the treatment of asthma. The major findings observed in this study are as follows. (1) A high ER protein folding requirement due to elevated PI3K–mTORC signaling and inflammation leads to ER stress and NF-κB-mediated stress responses, including proinflammatory cytokine expression, whereas a PI3Kδ inhibitor controls lung inflammation as well as the intra-ER accumulation of misfolded proteins and ROS. (2) The IRE-1α–RIDD–RIG-I axis was also regulated concomitantly with inhibition of downstream NF-κB signaling by administration of a PI3Kδ inhibitor. In addition to these mechanistic studies, we assessed the pharmacokinetics and bronchopulmonary disposition of the prototype PI3Kδ inhibitor IC87114.

This study utilized an established model of PI3K-induced asthma. The downstream mTORC1 (mammalian target of rapamycin complex 1) activity and protein synthesis status were shown to be regulated through PI3Kδ by a specific inhibitor, which also controlled the central inflammatory transcription factor NF-_K_B (Figures 2d, e). In the asthma condition, cell hyper-metabolism finally led to an excessive ER protein folding load, stimulating the UPR (Figure 1e). In this study, oxidative folding stress is discussed in the context of: (1) direct intra-ER hyperoxidation with a PDI-associated oxidizing protein status, (2) NOX4 involvement in intra-ER hyperoxidation and (3) a decreased ratio of reduced to oxidized GSH and the ensuing compromised ER thiol oxidative power. All three abnormalities were significantly reversed in a time-dependent manner by IC87114 treatment (Figures 3 and 4). Under excess protein folding conditions, the routine process of oxidation cannot be maintained. Considering that an oxidizing ER leads to a misfolding environment,^39^ our data suggest that PI3Kδ governs the flow of oxidizing equivalents to the ER thiol pool, allowing the cell to maintain ER redox conditions favorable for native disulfide formation. A single ER protein, PDI, catalyzes both the formation of disulfide bonds and disulfide exchange activities.^39^ Nox4 was shown to physically interact with PDI,^40^ and the downstream role of ROS generated by PDI−NOX could be related to ER-mediated phagocytosis^41^ and protein folding in the macrophage ER to affect antigen processing.^42^ ER stress-induced UPR signaling is associated with the production of many proinflammatory molecules.^43^ All three main branches of the UPR have been shown to mediate ‘cell autonomous’ proinflammatory transcriptional programs that are mainly governed by transcription factors, such as NF-κB.^44, 45^ Four NF-κB-associated inflammatory cytokines, IL-4, IL-5, IL-13 and IL-17, were highly upregulated in murine asthmatic lung tissue. Analysis of BALF further indicated that the present asthma model was characterized by sustained inflammation involving protein exudation, cell necrosis, recruitment of cells and cytokines (Figure 1b and Supplementary Figure 5a). These inflammatory cytokines are involved in the overproduction and overexpression of mucus proteins and genes in the airway epithelium.^46^ For example, T helper type 2 cytokines, such as IL-4, have been shown to regulate mucin gene expression.^47^ We observed hyperplasia and hypertrophy of airway goblet cells as well as peribronchial accumulation of inflammatory cells in mice following OVA/LPS treatment, responses that are clearly reduced by IC87114 (Figures 1f and 2f) Thus, the PI3K-associated high protein folding load and inflammatory status, leading to a hyperoxidative intra-ER environment and increased ER stress, can be controlled by PI3Kδ inhibition.

Among ER stress proteins, IRE1 was induced by inflammatory signaling through a pathway that includes RIDD and the antiviral RNA helicase RIG-I.^14, 15^ The RIG-I-like receptor double-stranded RNA helicase enzyme (RIG-I), a type of pattern recognition receptor, preferentially binds to short double-stranded RNAs and a 5′ceptor double-stranded RNA helicase to form large multimeric protein complexes with MAVS.^48^ These oligomeric complexes rapidly activate potent inflammatory responses through activation of NF-κB. The RIDD reaction produces single-strand mRNA fragments that activate RIG-I to cause a cell-autologous inflammatory response via the NF-κB and IFN pathways. In our model, the mRNA levels of IRE-1 RIDD substrates, including *Hqsnat*, *Blos1*, *Scara3*, *Pdqfrb*, *Pmp2* and *Col6*, were reduced (Figure 5b). In contrast, RIG-I as well as the three cytokines were robustly induced at the asthma exacerbation stage (Figures 5c–e), and these changes were controlled by IC87114. In addition to the PI3K-associated classical signaling pathway, mTORC1 and related protein hyperloading status, as well as RIG-I signaling activation by RIDD of IRE1α, were also suggested to promote the release of proinflammatory cytokines from lung epithelial cells.

In conclusion, sensitization and challenge with OVA/LPS resulted in the development of airway inflammation and remodeling, the severity of which was significantly reduced by the PI3Kδ inhibitor IC87114. More critically, the ER oxidative folding capacity was insufficient to maintain proper protein synthesis under this high immune activity, resulting in an abnormal PDI status, ER stress, exacerbation of inflammation, mucus hypersecretion and airway remodeling, all of which could be controlled by PI3Kδ inhibition. Moreover, IRE1α–RIDD–RIG1, another NF-κB-inflammation axis, was also controlled by IC87114. Thus, our data indicate that IC87114 may be an effective therapeutic agent for the treatment of severe asthma.

## References

[bib1] Leong KP, Huston DP. Understanding the pathogenesis of allergic asthma using mouse models. Ann Allergy Asthma Immunol 2001; 87: 96–109; quiz 110. 11527255 10.1016/S1081-1206(10)62201-6

[bib2] Kim YK, Oh SY, Jeon SG, Park HW, Lee SY, Chun EY et al. Airway exposure levels of lipopolysaccharide determine type 1 versus type 2 experimental asthma. J Immunol 2007; 178: 5375–5382. 17404323 10.4049/jimmunol.178.8.5375

[bib3] Eisenbarth SC, Piggott DA, Huleatt JW, Visintin I, Herrick CA, Bottomly K. Lipopolysaccharide-enhanced, toll-like receptor 4-dependent T helper cell type 2 responses to inhaled antigen. J Exp Med 2002; 196: 1645–1651. 12486107 10.1084/jem.20021340 PMC2196061

[bib4] Laird MH, Rhee SH, Perkins DJ, Medvedev AE, Piao W, Fenton MJ et al. TLR4/MyD88/PI3K interactions regulate TLR4 signaling. J Leukoc Biol 2009; 85: 966–977. 19289601 10.1189/jlb.1208763 PMC2698589

[bib5] Okkenhaug K, Vanhaesebroeck B. PI3K in lymphocyte development, differentiation and activation. Nat Rev Immunol 2003; 3: 317–330. 12669022 10.1038/nri1056

[bib6] Ali K, Bilancio A, Thomas M, Pearce W, Gilfillan AM, Tkaczyk C et al. Essential role for the p110delta phosphoinositide 3-kinase in the allergic response. Nature 2004; 431: 1007–1011. 15496927 10.1038/nature02991

[bib7] Lee KS, Lee HK, Hayflick JS, Lee YC, Puri KD. Inhibition of phosphoinositide 3-kinase delta attenuates allergic airway inflammation and hyperresponsiveness in murine asthma model. FASEB J 2006; 20: 455–465. 16507763 10.1096/fj.05-5045com

[bib8] Depuydt M, Leonard SE, Vertommen D, Denoncin K, Morsomme P, Wahni K et al. A periplasmic reducing system protects single cysteine residues from oxidation. Science 2009; 326: 1109–1111. 19965429 10.1126/science.1179557

[bib9] Boyle KB, Gyori D, Sindrilaru A, Scharffetter-Kochanek K, Taylor PR, Mocsai A et al. Class IA phosphoinositide 3-kinase beta and delta regulate neutrophil oxidase activation in response to Aspergillus fumigatus hyphae. J Immunol 2011; 186: 2978–2989. 21257963 10.4049/jimmunol.1002268

[bib10] Cao SS, Kaufman RJ. Endoplasmic reticulum stress and oxidative stress in cell fate decision and human disease. Antioxid Redox Signal 2014; 21: 396–413. 24702237 10.1089/ars.2014.5851 PMC4076992

[bib11] Wan WY, Hollins F, Haste L, Woodman L, Hirst RA, Bolton S et al. NADPH oxidase-4 overexpression is associated with epithelial ciliary dysfunction in neutrophilic asthma. Chest 2016; 149: 1445–1459. 26836936 10.1016/j.chest.2016.01.024 PMC4893823

[bib12] Kim SR, Kim DI, Kang MR, Lee KS, Park SY, Jeong JS et al. Endoplasmic reticulum stress influences bronchial asthma pathogenesis by modulating nuclear factor kappaB activation. J Allergy Clin Immunol 2013; 132: 1397–1408. 24161747 10.1016/j.jaci.2013.08.041

[bib13] Martinon F, Chen X, Lee AH, Glimcher LH. TLR activation of the transcription factor XBP1 regulates innate immune responses in macrophages. Nat Immunol 2010; 11: 411–418. 20351694 10.1038/ni.1857 PMC3113706

[bib14] Cho JA, Lee AH, Platzer B, Cross BC, Gardner BM, De Luca H et al. The unfolded protein response element IRE1alpha senses bacterial proteins invading the ER to activate RIG-I and innate immune signaling. Cell Host Microbe 2013; 13: 558–569. 23684307 10.1016/j.chom.2013.03.011 PMC3766372

[bib15] Eckard SC, Rice GI, Fabre A, Badens C, Gray EE, Hartley JL et al. The SKIV2L RNA exosome limits activation of the RIG-I-like receptors. Nat Immunol 2014; 15: 839–845. 25064072 10.1038/ni.2948 PMC4139417

[bib16] Lencer WI, DeLuca H, Grey MJ, Cho JA. Innate immunity at mucosal surfaces: the IRE1-RIDD-RIG-I pathway. Trends Immunol 2015; 36: 401–409. 26093676 10.1016/j.it.2015.05.006 PMC4490948

[bib17] Takeda M, Tanabe M, Ito W, Ueki S, Konnno Y, Chihara M et al. Gender difference in allergic airway remodelling and immunoglobulin production in mouse model of asthma. Respirology 2013; 18: 797–806. 23490273 10.1111/resp.12078

[bib18] Melgert BN, Postma DS, Kuipers I, Geerlings M, Luinge MA, van der Strate BW et al. Female mice are more susceptible to the development of allergic airway inflammation than male mice. Clin Exp Allergy 2005; 35: 1496–1503. 16297148 10.1111/j.1365-2222.2005.02362.x

[bib19] Kilkenny C, Browne W, Cuthill IC, Emerson M, Altman DG, NC3Rs Reporting Guidelines Working Group. Animal research: reporting *in vivo* experiments: the ARRIVE guidelines. Br J Pharmacol 2010; 160: 1577–1579. 20649561 10.1111/j.1476-5381.2010.00872.x PMC2936830

[bib20] McGrath JC, Lilley E. Implementing guidelines on reporting research using animals (ARRIVE etc.): new requirements for publication in BJP. Br J Pharmacol 2015; 172: 3189–3193. 25964986 10.1111/bph.12955 PMC4500358

[bib21] Rayamajhi M, Redente EF, Condon TV, Gonzalez-Juarrero M, Riches DW, Lenz LL. Non-surgical intratracheal instillation of mice with analysis of lungs and lung draining lymph nodes by flow cytometry. J Vis Exp 2011; 51: e2702. 10.3791/2702 PMC3280633 21587154

[bib22] Park SJ, Lee KS, Kim SR, Min KH, Moon H, Lee MH et al. Phosphoinositide 3-kinase delta inhibitor suppresses interleukin-17 expression in a murine asthma model. Eur Respir J 2010; 36: 1448–1459. 20351038 10.1183/09031936.00106609

[bib23] Bates JH, Irvin CG. Measuring lung function in mice: the phenotyping uncertainty principle. J Appl Physiol 19852003; 94: 1297–1306. 10.1152/japplphysiol.00706.2002 12626466

[bib24] Shen HH, Ochkur SI, McGarry MP, Crosby JR, Hines EM, Borchers MT et al. A causative relationship exists between eosinophils and the development of allergic pulmonary pathologies in the mouse. J Immunol 2003; 170: 3296–3305. 12626589 10.4049/jimmunol.170.6.3296

[bib25] Kim HR, Lee GH, Ha KC, Ahn T, Moon JY, Lee BJ et al. Bax inhibitor-1 is a pH-dependent regulator of Ca^2+^ channel activity in the endoplasmic reticulum. J Biol Chem 2008; 283: 15946–15955. 18378668 10.1074/jbc.M800075200 PMC2414309

[bib26] Kenche H, Baty CJ, Vedagiri K, Shapiro SD, Blumental-Perry A. Cigarette smoking affects oxidative protein folding in endoplasmic reticulum by modifying protein disulfide isomerase. FASEB J 2013; 27: 965–977. 23169770 10.1096/fj.12-216234

[bib27] Lee S, Min Kim S, Dotimas J, Li L, Feener EP, Baldus S et al. Thioredoxin-interacting protein regulates protein disulfide isomerases and endoplasmic reticulum stress. EMBO Mol Med 2014; 6: 732–743. 24843047 10.15252/emmm.201302561 PMC4203352

[bib28] Zito E, Hansen HG, Yeo GS, Fujii J, Ron D. Endoplasmic reticulum thiol oxidase deficiency leads to ascorbic acid depletion and noncanonical scurvy in mice. Mol Cell 2012; 48: 39–51. 22981861 10.1016/j.molcel.2012.08.010 PMC3473360

[bib29] Aguilera-Aguirre L, Bacsi A, Radak Z, Hazra TK, Mitra S, Sur S et al. Innate inflammation induced by the 8-oxoguanine DNA glycosylase-1-KRAS-NF-kappaB pathway. J Immunol 2014; 193: 4643–4653. 25267977 10.4049/jimmunol.1401625 PMC4201976

[bib30] Long X, Li S, Xie J, Li W, Zang N, Ren L et al. MMP-12-mediated by SARM-TRIF signaling pathway contributes to IFN-gamma-independent airway inflammation and AHR post RSV infection in nude mice. Respir Res 2015; 16: 11. 25652021 10.1186/s12931-015-0176-8 PMC4332892

[bib31] Curtis MJ, Bond RA, Spina D, Ahluwalia A, Alexander SP, Giembycz MA et al. Experimental design and analysis and their reporting: new guidance for publication in BJP. Br J Pharmacol 2015; 172: 3461–3471. 26114403 10.1111/bph.12856 PMC4507152

[bib32] Henke MO, John G, Germann M, Lindemann H, Rubin BK. MUC5AC and MUC5B mucins increase in cystic fibrosis airway secretions during pulmonary exacerbation. Am J Respir Crit Care Med 2007; 175: 816–821. 17255563 10.1164/rccm.200607-1011OC

[bib33] Zuhdi Alimam M, Piazza FM, Selby DM, Letwin N, Huang L, Rose MC. Muc-5/5ac mucin messenger RNA and protein expression is a marker of goblet cell metaplasia in murine airways. Am J Respir Cell Mol Biol 2000; 22: 253–260. 10696060 10.1165/ajrcmb.22.3.3768

[bib34] Nashed BF, Zhang T, Al-Alwan M, Srinivasan G, Halayko AJ, Okkenhaug K et al. Role of the phosphoinositide 3-kinase p110delta in generation of type 2 cytokine responses and allergic airway inflammation. Eur J Immunol 2007; 37: 416–424. 17236236 10.1002/eji.200636401

[bib35] Ciencewicki J, Trivedi S, Kleeberger SR. Oxidants and the pathogenesis of lung diseases. J Allergy Clin Immunol 2008; 122: 456–468; quiz 469–470. 18774381 10.1016/j.jaci.2008.08.004 PMC2693323

[bib36] Dalle-Donne I, Rossi R, Giustarini D, Milzani A, Colombo R. Protein carbonyl groups as biomarkers of oxidative stress. Clin Chim Acta 2003; 329: 23–38. 12589963 10.1016/s0009-8981(03)00003-2

[bib37] Zeeshan HM, Lee GH, Kim HR, Chae HJ. Endoplasmic reticulum stress and associated ROS. Int J Mol Sci 2016; 17: 327. 26950115 10.3390/ijms17030327 PMC4813189

[bib38] Mahmutovic Persson I, Akbarshahi H, Menzel M, Brandelius A, Uller L. Increased expression of upstream TH2-cytokines in a mouse model of viral-induced asthma exacerbation. J Transl Med 2016; 14: 52. 26879906 10.1186/s12967-016-0808-x PMC4754855

[bib39] Gilge JL, Fisher M, Chai YC. The effect of oxidant and the non-oxidant alteration of cellular thiol concentration on the formation of protein mixed-disulfides in HEK 293 cells. PLoS ONE 2008; 3: e4015. 19107210 10.1371/journal.pone.0004015 PMC2603474

[bib40] Janiszewski M, Lopes LR, Carmo AO, Pedro MA, Brandes RP, Santos CX et al. Regulation of NAD(P)H oxidase by associated protein disulfide isomerase in vascular smooth muscle cells. J Biol Chem 2005; 280: 40813–40819. 16150729 10.1074/jbc.M509255200

[bib41] Gordon S. Alternative activation of macrophages. Nat Rev Immunol 2003; 3: 23–35. 12511873 10.1038/nri978

[bib42] Santos CX, Nabeebaccus AA, Shah AM, Camargo LL, Filho SV, Lopes LR. Endoplasmic reticulum stress and Nox-mediated reactive oxygen species signaling in the peripheral vasculature: potential role in hypertension. Antioxid Redox Signal 2014; 20: 121–134. 23472786 10.1089/ars.2013.5262 PMC3880927

[bib43] Li Y, Schwabe RF, DeVries-Seimon T, Yao PM, Gerbod-Giannone MC, Tall AR et al. Free cholesterol-loaded macrophages are an abundant source of tumor necrosis factor-alpha and interleukin-6: model of NF-kappaB- and map kinase-dependent inflammation in advanced atherosclerosis. J Biol Chem 2005; 280: 21763–21772. 15826936 10.1074/jbc.M501759200

[bib44] Verfaillie T, Garg AD, Agostinis P, Targeting ER. stress induced apoptosis and inflammation in cancer. Cancer Lett 2013; 332: 249–264. 20732741 10.1016/j.canlet.2010.07.016

[bib45] Hotamisligil GS, Erbay E. Nutrient sensing and inflammation in metabolic diseases. Nat Rev Immunol 2008; 8: 923–934. 19029988 10.1038/nri2449 PMC2814543

[bib46] Lee C, Kolesnik TB, Caminschi I, Chakravorty A, Carter W, Alexander WS et al. Suppressor of cytokine signalling 1 (SOCS1) is a physiological regulator of the asthma response. Clin Exp Allergy 2009; 39: 897–907. 19309352 10.1111/j.1365-2222.2009.03217.x PMC3449009

[bib47] Karras JG, Crosby JR, Guha M, Tung D, Miller DA, Gaarde WA et al. Anti-inflammatory activity of inhaled IL-4 receptor-alpha antisense oligonucleotide in mice. Am J Respir Cell Mol Biol 2007; 36: 276–285. 16990616 10.1165/rcmb.2005-0456OC

[bib48] Reikine S, Nguyen JB, Modis Y. Pattern recognition and signaling mechanisms of RIG-I and MDA5. Front Immunol 2014; 5: 342. 25101084 10.3389/fimmu.2014.00342 PMC4107945

