# Supplementary material for: PI3Kδ contributes to ER stress-associated asthma through ER-redox disturbances: the involvement of the RIDD–RIG-I–NF-κB axis
Source: Exp Mol Med. 2018 Feb 16;50(2):e444–. doi: 10.1038/emm.2017.270 (PMC5903822; doi:10.1038/emm.2017.270)

**PI3Kδ contributes to ER stress-associated asthma through ER-redox disturbances; the involvement of RIDD-RIG-1 -NF-κB axis**

Hyun-Kyoung Kim^1^, Geum-Hwa Lee^1^, Kashi Raj Bhattarai^1^, Raghu Patil Junjappa^1^, Hwa-Young Lee^1^, Mallikarjun Handigund^1^, Anu Marahatta^1^, Bidur Bhandary^1^, In-hwan Baek^2^, Jae Sung Pyo^2^, Hye-Kyung Kim^2^, Ok Hee Chai^3^, Hyung-Ryong Kim^4^, Yong-Chul Lee^5^ & Han-Jung Chae^1^

^1^Department of Pharmacology and Institute of New Drug Development, School of Medicine, Chonbuk National University, Jeonju; ^2^College of Pharmacy, Kyungsung University, Busan; ^3^Department of Anatomy, School of Medicine, Chonbuk National University, Jeonju;^4^Daegu Gyeonbuk Institute of Science & Technology (DGIST) graduate school, Daegu, Republic of Korea; ^5^Department of Internal Medicine, School of Medicine, Chonbuk National University, Jeonju;

**Correspondence to** Professor Han-Jung Chae, Ph.D., Department of Pharmacology and Institute of Cardiovascular Research, Medical School, Chonbuk National University, Jeonju, 54896, Republic of Korea. Tel: +82-63-270-3092, Fax: +82-63-275-2855; E-mail: [hjchae@chonbuk.ac.kr](mailto:hjchae@chonbuk.ac.kr)

Current address: Bidur Bhandary, PhD, Molecular Cardiovascular Division, The Heart Institute; Anu Marahatta, PhD, Hematology Department, Cincinnati Childrens Hospital Medical Center, Cincinnati, OH

**Supplementary Figure 1. Experimental design and time course of bronchoalveolar lavage inflammatory cell infiltration.** (A) Mice were sensitized intranasally (i.n) with OVA plus LPS on days 0, 1, 2, 3, and 7, and then challenged intranasally with OVA alone on days 14, 15, 21, and 22. On day 22 (3 h after the last airway challenge with OVA) IC87114 at 1 mg/kg or vehicle (0.05% DMSO) diluted in 0.9% NaCl was administered in a volume of 30 μl by an intratracheal nonsurgical method. (B) Immunoblotting and densitometric analyses of NF-κB and IκBα proteins from the cytosol fraction of samples collected by subcellular fraction of lung tissues. Immunoblotting of GRP78, CHOP, ATF-6α, p-PERK, PERK, p-eIF2α, eIF2α, p-IRE-1α, IRE-1α, sXBP-1, and β-actin performed as described in Methods (C) and densitometric analyses of p-PERK/PERK, p-eIF2α/eIF2α, ATF-6α, and sXBP-1 (D). Values shown are the mean ± SEM (*n* =6). The mice treated with saline (SAL) were used as control.

**Supplementary Figure 2. Time course of bronchoalveolar lavage inflammatory cell infiltration.** OVA/LPS-induced asthmatic mice were treated with IC87114 at 1 mg⋅kg-1 and BALF cells (A) and lung tissue (B) were obtained at the indicated post-treatment time points and stained with Diff-Quick solution and hematoxylin-eosin, respectively.

**Supplementary Figure 3. Effects of IC87114 on differential cell counts of bronchoalveolar lavage fluid.** Total cell counts (A) and differential cell count (B and C) were performed to identify neutrophil count (B) and eosinophil count (C). (D) Airway reactivity in response to increasing doses of nebulized methacholine was assessed by whole-body plethysmography. Each point represents the mean ± SEM (*n* =6). The mice treated with saline (SAL) were used as control. #p < 0.05 versus SAL; *p < 0.05 versus OVA/LPS only.

**Supplementary Figure 4. Effects of IC87114 on PI3K downstream effector proteins in lung tissues.** OVA/LPS-induced asthmatic mice were treated with IC87114 at 1 mg/kg. After 24 hours, lung tissue were immunostained for p4E-BP1 and p-S6K as described in Materials and Methods.

**Supplementary Figure 5. Effects of IC87114 on cytokine levels in bronchoalveolar lavage fluid.** Mice were treated as described in Supplementary Figure 1A. BALF was collected at different time points and then analyzed by ELISA for production of the cytokines IL-4, IL-5, IL-13, and IL-17 in vehicle treatment to OVA/LPS induced mice (A), or IL-4 (B), IL-5 (C), IL-13 (D), and IL-17 (E) in IC87114 treatment to OVA/LPS induced mice. Values shown are the mean ± SEM (*n* = 6). The mice treated with saline (SAL) were used as control.. #p < 0.05 versus SAL; *p < 0.05 versus OVA/LPS only.

**Supplementary Figure 6. Effects of IC87114 on mRNA levels in lung tissues.** Lung samples were collected and total RNA was extracted. Real time PCR was performed to examine the relative levels of IL-4, IL-5, IL-13, and IL-17 mRNA in vehicle treatment to OVA/LPS induced mice (A),IL-4 (B), IL-5 (C), IL-13 (D), and IL-17 (E) in IC87114 treatment to OVA/LPS induced mice. Values shown are the mean ± SEM (*n* = 6). The mice treated with saline (SAL) were used as control. #p < 0.05 versus SAL; *p < 0.05 versus OVA/LPS only.

**Supplementary Figure 7. Effects of NF-**κ**B siRNA treatment *in vivo.*** Mice were treated as described in the Materials and Methods. (A) Total cell counts were performed in BALF. (B) Immunoblotting of NF-κB proteins from the cytosol and nuclear fractions collected by subcellular fractionation of lung tissues were performed. (C) BALF was collected and then was analyzed by ELISA for production of the cytokines IL-4, IL-5, and IL-13 in NF-κB siRNA-treated mice. Values shown are the mean ± SEM (*n* = 6). *p < 0.05 versus control siRNA.

**Supplementary Figure 8. OVA plus LPS induces ROS accumulation in ER.** (A) 4-HNE staining in lung tissues from OVA/LPS, SAL, and OVA/LPS-treated mice administered vehicle (0.05% DMSO)). (B) Lysates from lung tissues were analyzed for the presence of oxidized proteins by oxyblot analysis. (C) Hydrogen peroxide levels were measured in lung ER fractions. (D) Heavy molecular weight complex formation was analyzed with anti-PDI antibody. Values shown are means ± SEM (*n* = 6). The mice treated with saline (SAL) were used as control. #p < 0.05 versus SAL.

**Supplementary Figure 9. Recovery effects of PDI redox*.*** Lung samples were collected and lysates were treated with 1 mM diamide for 15 min followed by treatment with the indicated concentrations of DTT. Immunoblotting (upper) and quantification (lower) of the reduced and oxidized forms of PDI were analyzed. Values shown are the mean ± SEM. #p < 0.05 versus SAL; *p < 0.05 versus OVA/LPS.

**Supplementary Figure 10. Effects of** IC87114 on ER stress response. Immunoblotting of GRP78, CHOP, ATF-6α, p-PERK, PERK, p-eIF2α, eIF2α, p-IRE-1α, IRE-1α, sXBP-1, and β-actin (performed as described in Methods). The mice treated with saline (SAL) were used as control.

**Supplementary Figure 11. Effects of** IC87114 on *sXBP1*. RT-PCR for *Xbp-1* at the indicated time in lung tissues from OVA/LPS, SAL-treated mice, and OVA/LPS-treated mice administered vehicle (0.05% DMSO). *uXbp-1*, unspliced forms of *Xbp-1* mRNA; *sXbp-1*, spliced forms of *Xbp-1* mRNA; The mice treated with saline (SAL) were used as control.

Table S1. Murine primer sequences for genes analyzed by RT-qPCR.

| Gene | Forward | Reverse |
| --- | --- | --- |
| *IL-4* | TCATCGGCATTTTGAACGAG | CGTTTGGCACATCCATCTCC |
| *IL-5* | AAAGAGAAGTGTGGCGAGGAGA | CACCAAGGAACTCTTGCAGGTAA |
| *IL-13* | GAGCAACATCACACAAGACCAGA | GGCCAGGTCCACACTCCATA |
| *IL-17* | TCTCATCCAGCAAGAGATCC | AGTTTGGGACCCCTTTACAC |
| *XBP-1* | GAGTCCGCAGCAGGTG | GTGTCAGAGTCCATGGGA |
| *IL-33* | CAATGTTGACGACTCTGGAAAAG | GGGACTCATGTTCACCATCAG |
| *TSLP* | AAACTGAGAGAAATGACGGTACT | TCTGGAGATTGCATGAAGGAATA |
| *IL-25* | CGGAGGAGTGGCTGAAGTGGAG | ATGGGTACCTTCCTCGCCATG |
| *RIG-1* | TTGCTGAGTGCAATCTCGTC | GTATGCGGTGAACCGTCTTT |
| *NOX4* | TCCAAGCTCATTTCCCACAG | CGGAGTTCCATTACATCAGAGG |
| *Hprt* | GCTGGTGAAAAGGACCTCTGC | CACAGGACTAGAACACCTGC |
| *Hgsnat* | TCTCCGCTTTCTCCATTTTG | CGCATACACGTGGAAAGTCA |
| *Blos1* | CAAGGAGCTGCAGGAGAAGA | GCCTGGTTGAAGTTCTCCAC |
| *Scara3* | TGCATGGATACTGACCCTGA | GCCGTGTTACCAGCTTCTTC |
| *Pdgfrb* | AACCCCCTTACAGCTGTCCT | TAATCCCGTCAGCATCTTCC |
| *Pmp22* | TGCGATACAGCAGAATGGAG | TTGGTGGCCAATACAAGTCA |
| *Col6* | TGCTCAACATGAAGCAGACC | TTGAGGGAGAAAGCTCTGGA |
| *Gapdh* | TTCACCACCATGGAGAAGGC | GGCATGGACTGTGGTCATGA |


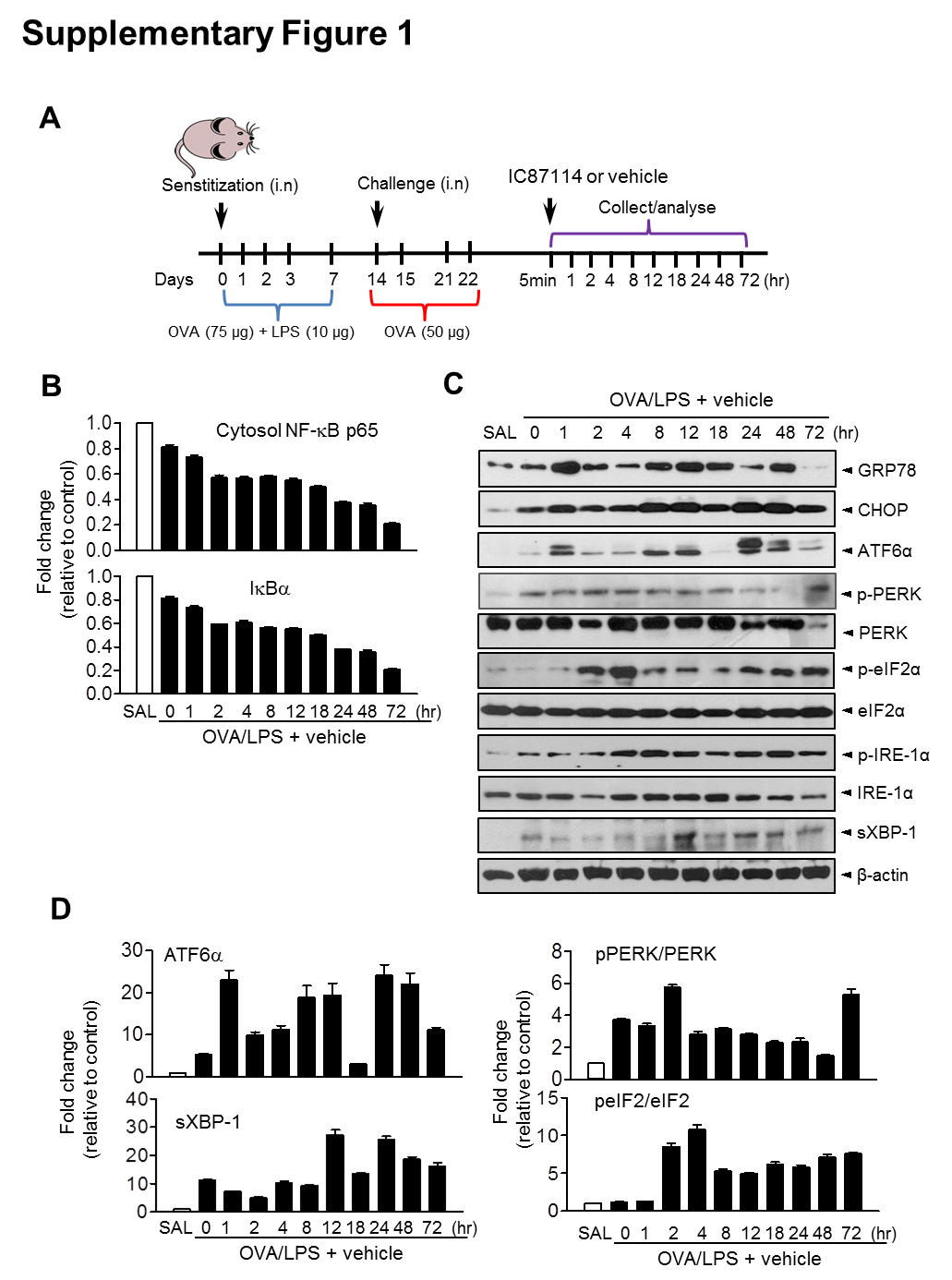


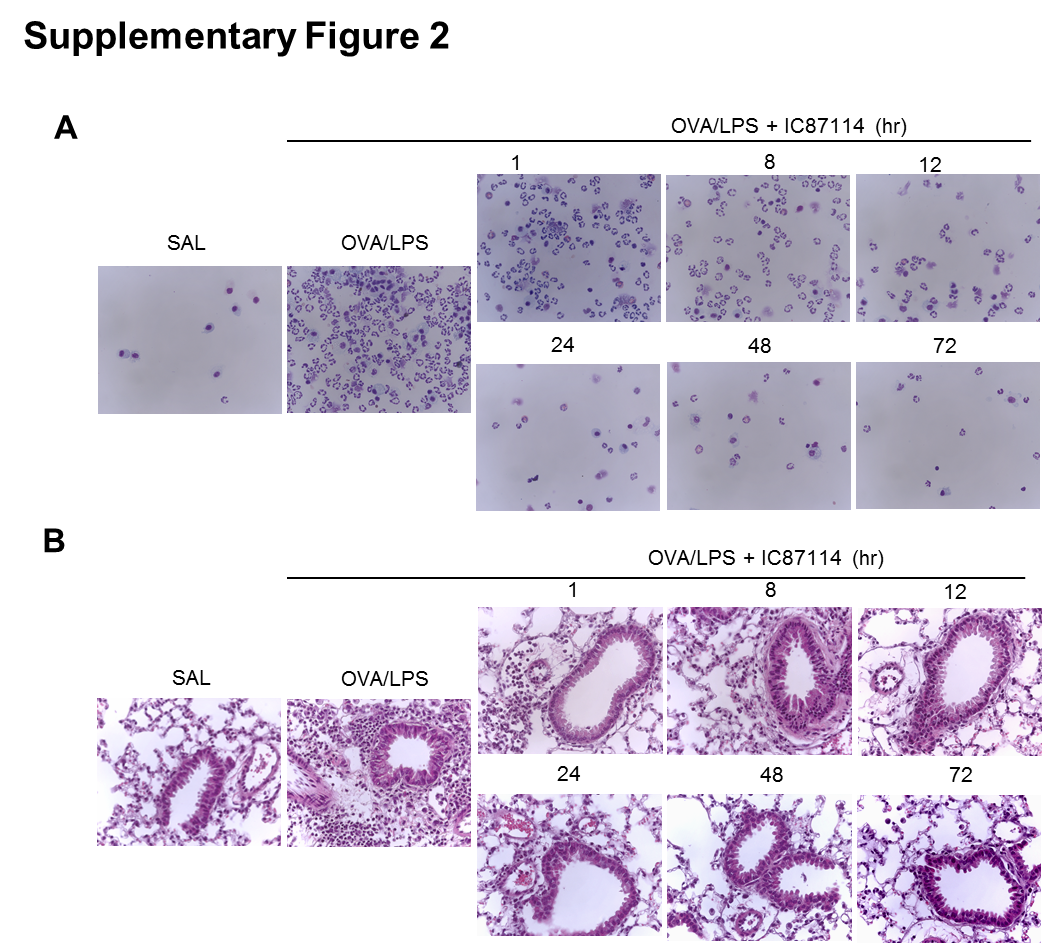


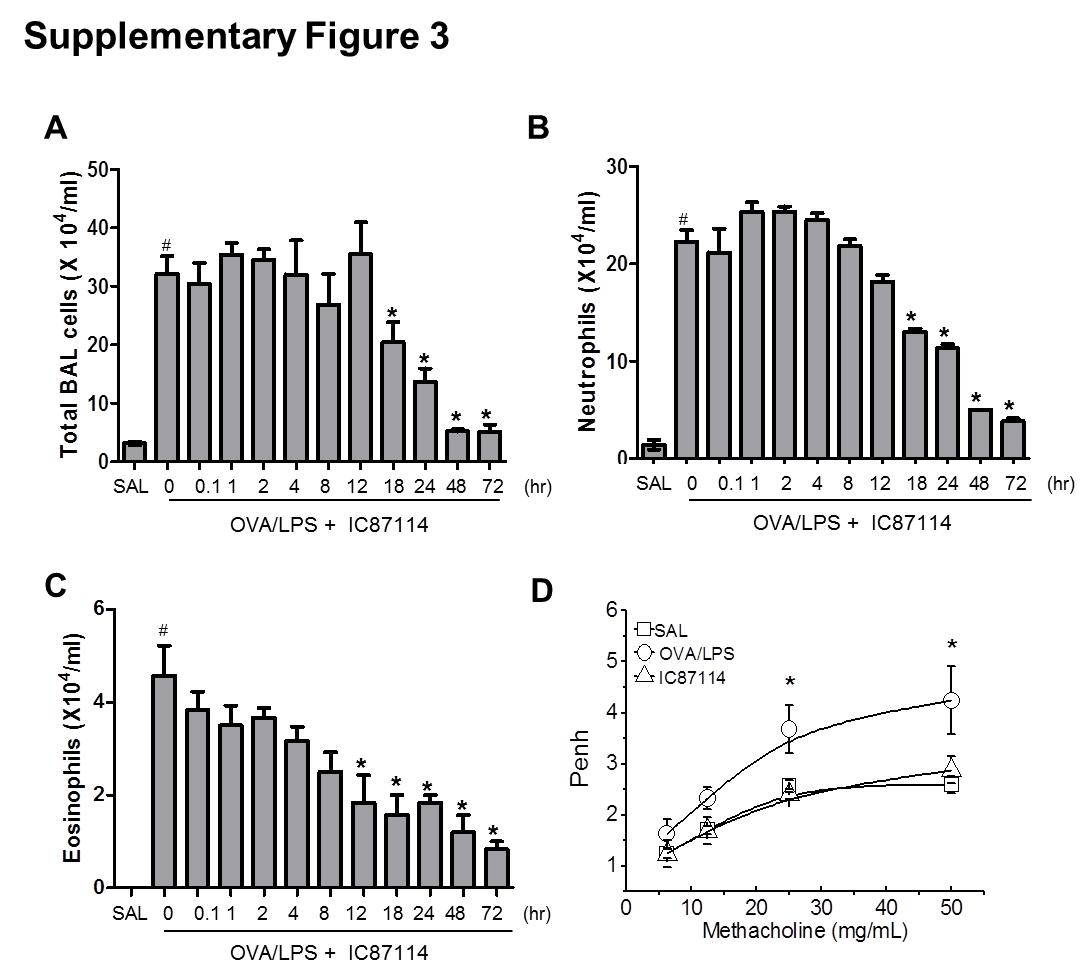


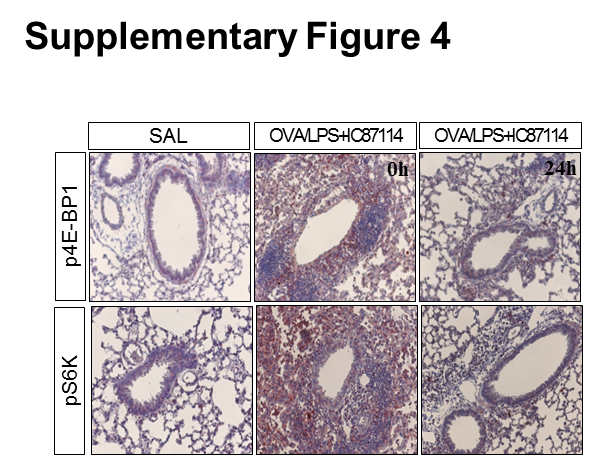


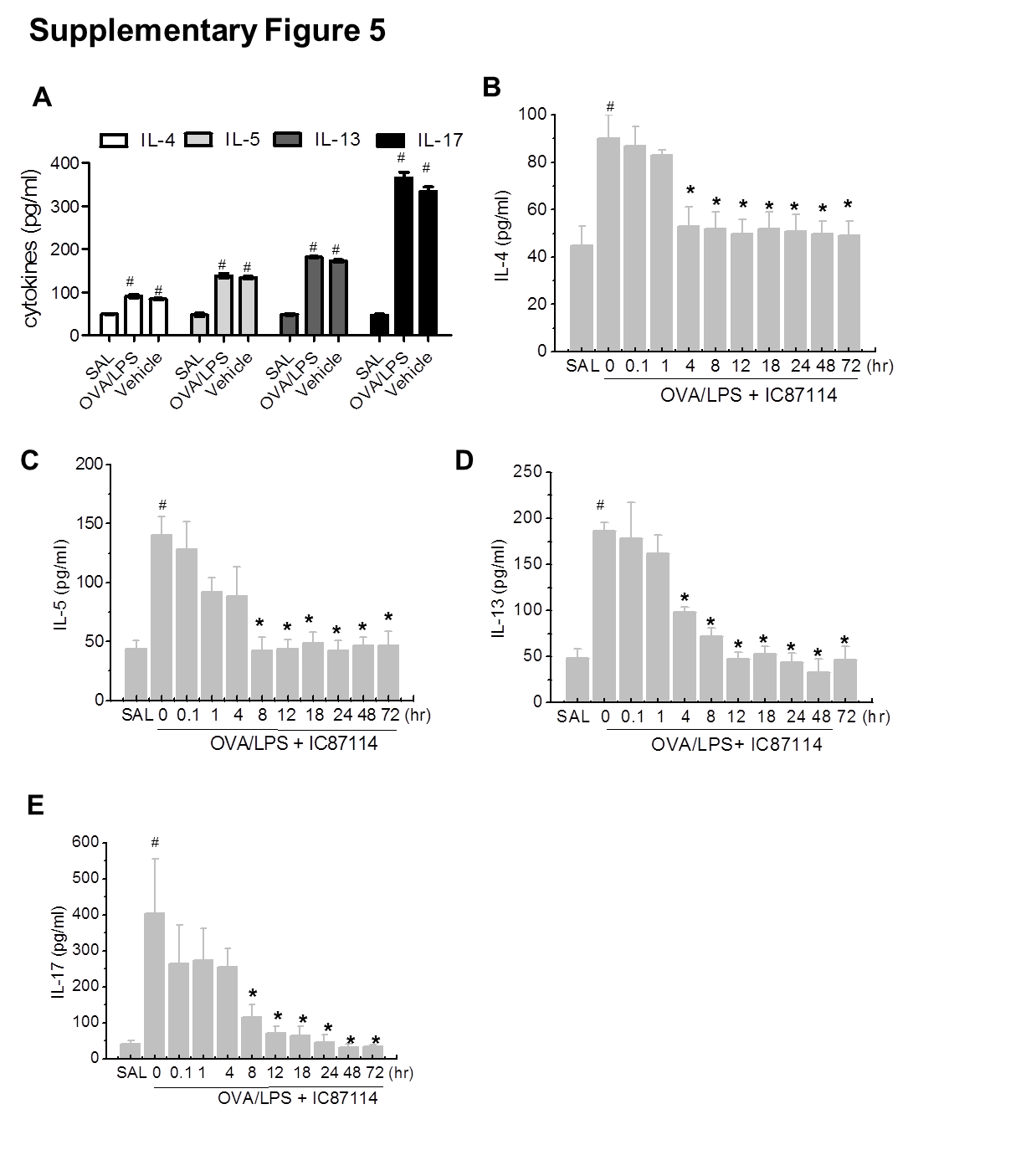


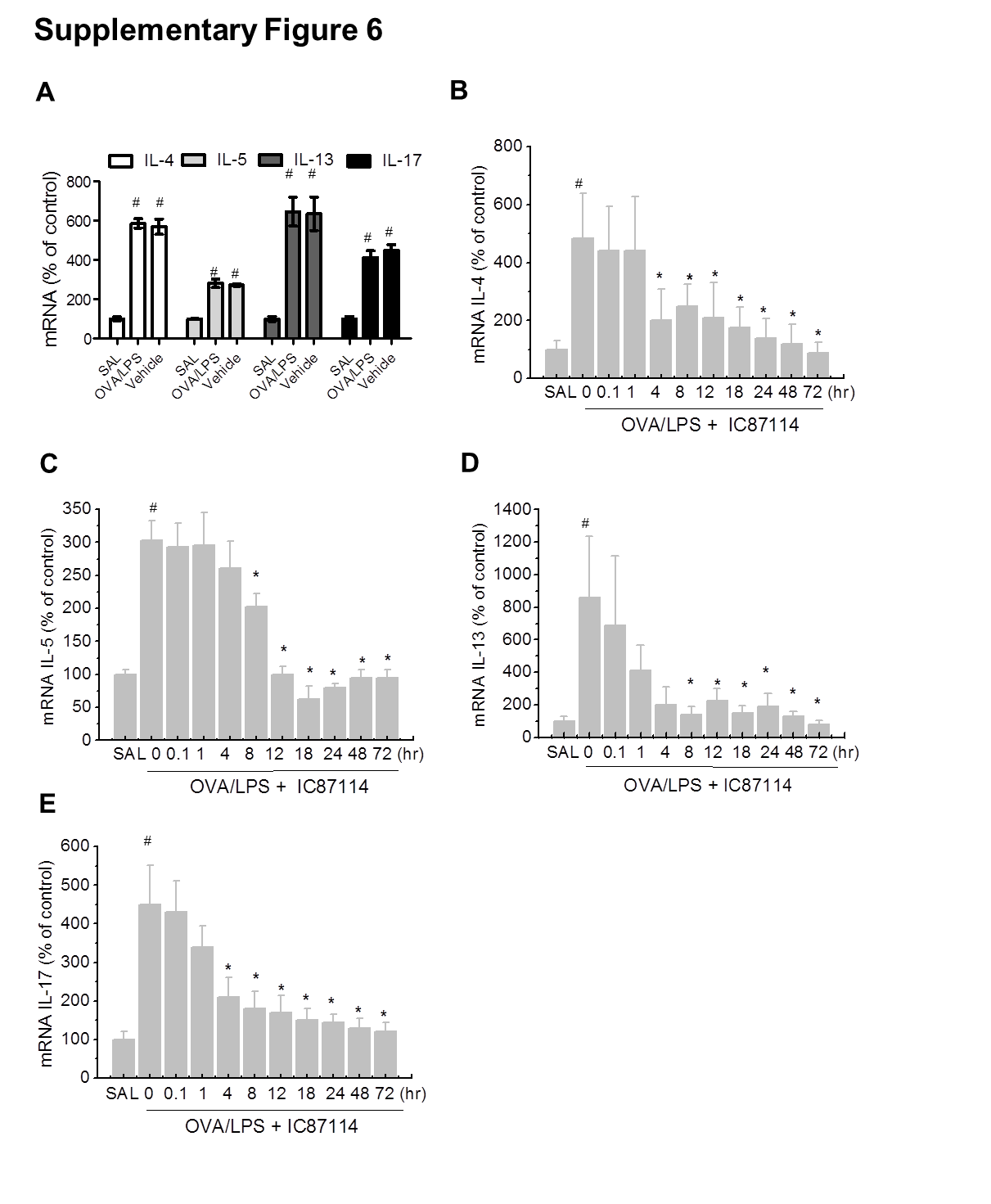


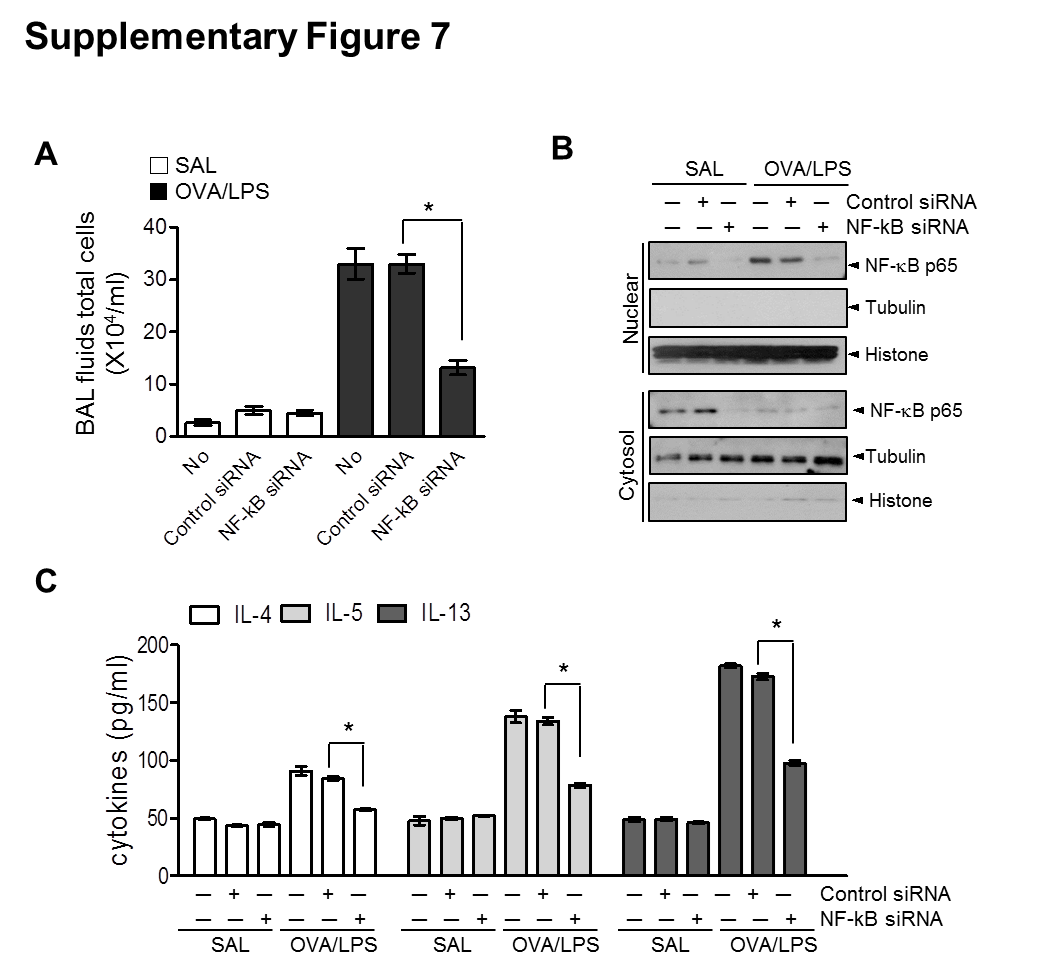


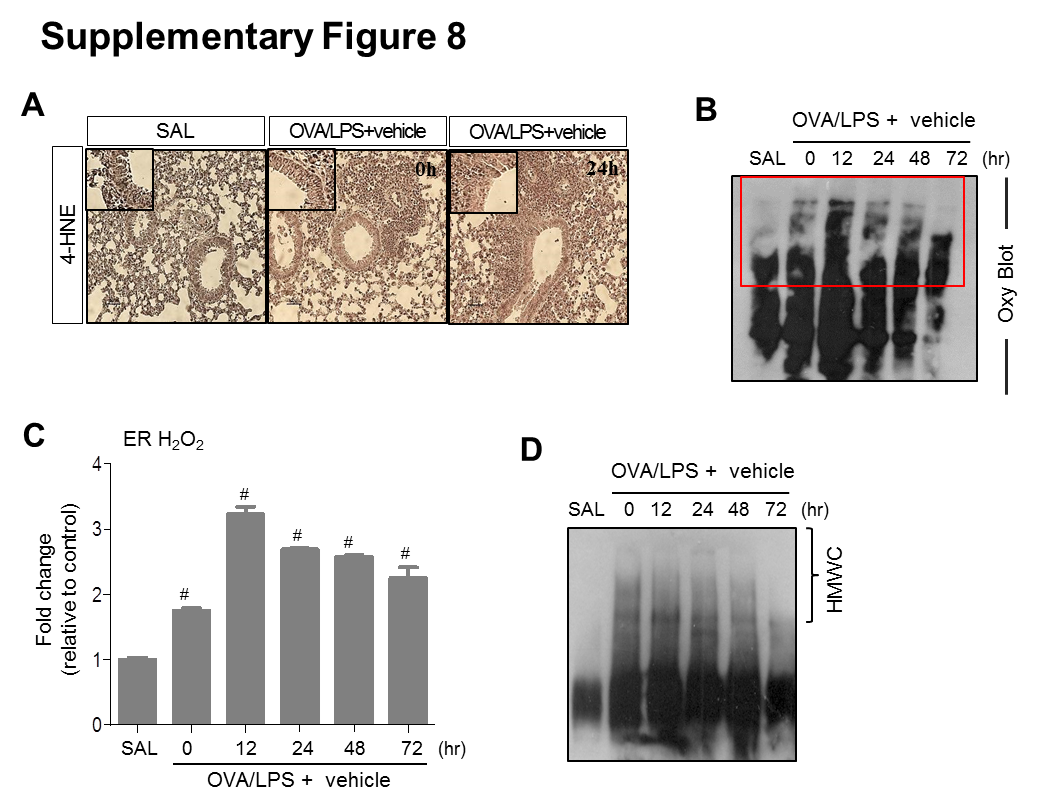


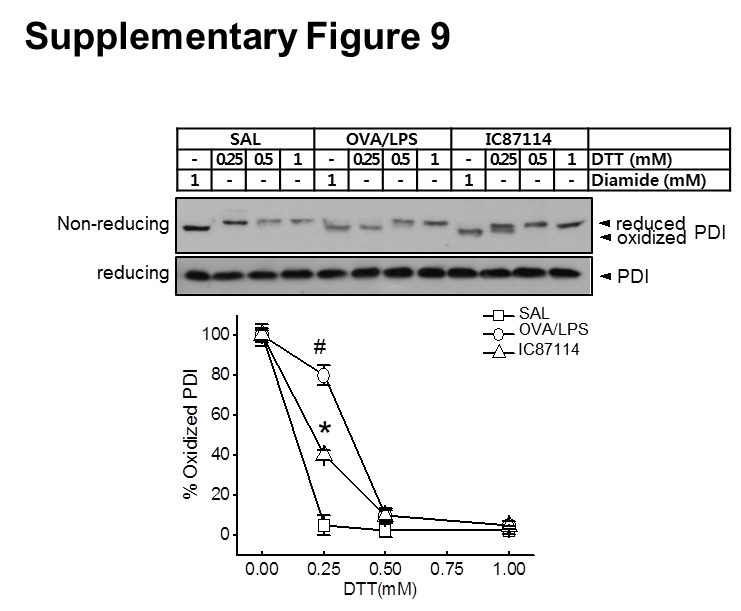


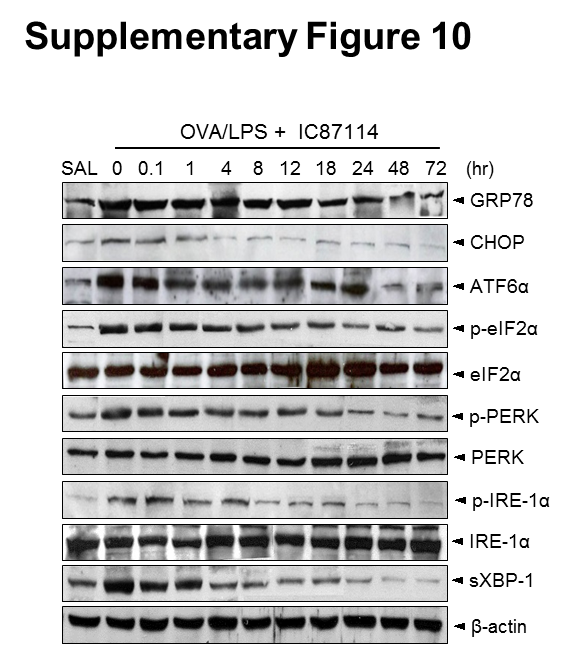


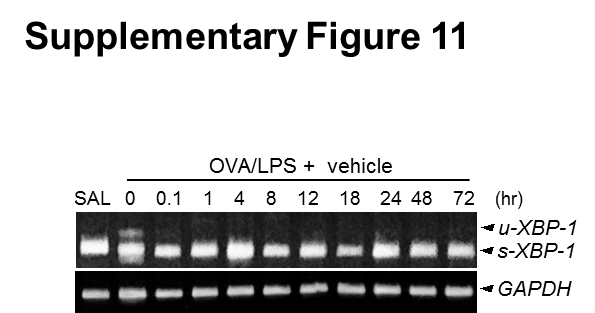

Supplement: Supplementary Information [file emm2017270x1.docx]
